# An Explainable Artificial Intelligence Text Classifier for Suicidality Prediction in Youth Crisis Text Line Users: Development and Validation Study

**DOI:** 10.2196/63809

**Published:** 2025-01-29

**Authors:** Julia Thomas, Antonia Lucht, Jacob Segler, Richard Wundrack, Marcel Miché, Roselind Lieb, Lars Kuchinke, Gunther Meinlschmidt

**Affiliations:** 1 Division of Clinical Psychology and Epidemiology Faculty of Psychology University of Basel Basel Switzerland; 2 Division of Clinical Psychology and Cognitive Behavioural Therapy International Psychoanalytic University Berlin Berlin Germany; 3 Department of Research, Analytics and Development krisenchat gGmbH Berlin Germany; 4 Division of Child and Adolescent Psychiatry/Psychotherapy Universitätsklinikum Ulm Ulm Germany; 5 Division of Methods and Statistics International Psychoanalytic University Berlin Berlin Germany; 6 Clinical Psychology and Psychotherapy Methods and Approaches, Department of Psychology Trier University Trier Germany; 7 Department of Digital and Blended Psychosomatics and Psychotherapy, Psychosomatic Medicine University Hospital and University of Basel Basel Switzerland; 8 Department of Psychosomatic Medicine University Hospital Basel University of Basel Basel Switzerland

**Keywords:** deep learning, explainable artificial intelligence (XAI), large language model (LLM), machine learning, neural network, prevention, risk monitoring, suicide, transformer model, suicidality, suicidal ideation, self-murder, self-harm, youth, adolescent, adolescents, public health, language model, language models, chat protocols, crisis helpline, help-seeking behaviors, German, Shapley, decision-making, mental health, health informatics, mobile phone

## Abstract

**Background:**

Suicide represents a critical public health concern, and machine learning (ML) models offer the potential for identifying at-risk individuals. Recent studies using benchmark datasets and real-world social media data have demonstrated the capability of pretrained large language models in predicting suicidal ideation and behaviors (SIB) in speech and text.

**Objective:**

This study aimed to (1) develop and implement ML methods for predicting SIBs in a real-world crisis helpline dataset, using transformer-based pretrained models as a foundation; (2) evaluate, cross-validate, and benchmark the model against traditional text classification approaches; and (3) train an explainable model to highlight relevant risk-associated features.

**Methods:**

We analyzed chat protocols from adolescents and young adults (aged 14-25 years) seeking assistance from a German crisis helpline. An ML model was developed using a transformer-based language model architecture with pretrained weights and long short-term memory layers. The model predicted suicidal ideation (SI) and advanced suicidal engagement (ASE), as indicated by composite Columbia-Suicide Severity Rating Scale scores. We compared model performance against a classical word-vector-based ML model. We subsequently computed discrimination, calibration, clinical utility, and explainability information using a Shapley Additive Explanations value-based post hoc estimation model.

**Results:**

The dataset comprised 1348 help-seeking encounters (1011 for training and 337 for testing). The transformer-based classifier achieved a macroaveraged area under the curve (AUC) receiver operating characteristic (ROC) of 0.89 (95% CI 0.81-0.91) and an overall accuracy of 0.79 (95% CI 0.73-0.99). This performance surpassed the word-vector-based baseline model (AUC-ROC=0.77, 95% CI 0.64-0.90; accuracy=0.61, 95% CI 0.61-0.80). The transformer model demonstrated excellent prediction for nonsuicidal sessions (AUC-ROC=0.96, 95% CI 0.96-0.99) and good prediction for SI and ASE, with AUC-ROCs of 0.85 (95% CI 0.97-0.86) and 0.87 (95% CI 0.81-0.88), respectively. The Brier Skill Score indicated a 44% improvement in classification performance over the baseline model. The Shapley Additive Explanations model identified language features predictive of SIBs, including self-reference, negation, expressions of low self-esteem, and absolutist language.

**Conclusions:**

Neural networks using large language model–based transfer learning can accurately identify SI and ASE. The post hoc explainer model revealed language features associated with SI and ASE. Such models may potentially support clinical decision-making in suicide prevention services. Future research should explore multimodal input features and temporal aspects of suicide risk.

## Introduction

Suicide, the third leading cause of premature mortality among German adolescents [1], encompasses a spectrum from suicidal ideation (SI) to preparatory actions [2-5]. Adolescents’ vulnerability to suicidal phenomena stems from a complex interplay of biological, genetic, psychological, and social factors [6-9]. Alarmingly, less than 50% of adolescents who attempt suicide receive appropriate psychiatric intervention [10]. This gap between need and care emphasizes the critical importance of accurately identifying and timely identification of at-risk individuals, challenging mental health care providers and educational institutions.

The digital age, characterized by the frequent use of the internet and smartphones, has transformed youth help-seeking behaviors, with online text-based services becoming the preferred communication mode [11-13]. These platforms align with young people’s digital environments [12,14], reducing help-seeking barriers such as limited service availability and stigma [15]. Their immediacy and anonymity are crucial for crisis help-seeking, establishing text-based helplines as critical public health measures for suicide-related issues [15,16]. While presenting challenges for clinicians in rapidly assessing risks and implementing interventions [17], these digitized services also offer opportunities. They enable traditional clinical risk monitoring models [18] to be complemented or potentially superseded by machine learning (ML) approaches.

ML has shown promise in identifying risk factors associated with suicide risk [19-21] and suicidal outcomes, including SI [22], behaviors [21,23], attempts [24], and completed suicides [25,26]. ML models use various input data types, from electronic health records [14,24,27] to textual data [19,28], collected in diverse settings [12,19,24,28,29]. These “theory-free” approaches [30] often outperform traditional detection methods [31].

Natural language processing advancements, particularly pretrained large language models based on transformer architectures, have significantly enhanced language classification tasks [32,33]. These “foundational models” [34], such as BERT, RoBERTa, LLaMA, and GPT, provide probabilistic language representations applicable to diverse tasks, opening new avenues for ML-based prediction in language-based psychological domains [34].

Language models provide rich text embeddings for transfer learning and fine-tuning [35,36], potentially enabling more accurate detection of SI and behaviors, even with limited domain-specific data [37]. Recent research has highlighted ML techniques, particularly foundational language models, in comprehensive suicidality prediction [38,39] and extraction of relevant textual indicators [40,41]. Transformer-based models outperform traditional ML approaches in suicide prediction using textual data, especially in social media datasets [38,42-45], though their real-world clinical applicability has remained unexplored.

The clinical implementation of ML models requires rigorous validation [46]. The “black box” nature of ML decisions necessitates explainable AI to emphasize interpretability for clinicians [30,31]. While traditional rule-based ML approaches such as decision trees offer straightforward explainability, the complexity of deep neural networks in foundational models presents interpretability challenges [47]. Model-agnostic post hoc explanation approaches, such as Shapley Additive Explanations (SHAPs), address this complexity [48-50], potentially elucidating key language features associated with suicide risk.

Deep neural network models have been applied to social media data [29,51-56], and explainable AI studies have been conducted on medical tabular data [57] and electronic health records [58]. However, studies using textual data from clinical populations, particularly helpline data, have not adequately addressed explainability.

Clinical implementations of ML as a diagnostic tool require rigor and transparency beyond interpretability. Ensuring model stability, tuning, and clinical value is crucial when considering the cost-benefit ratio of diagnostic decisions [59,60]. Metrics such as calibration and clinical utility [61-64] can emphasize the reliability and practical value of ML-based decisions in suicide risk prediction, though they remain underused in this context.

Our study aims to bridge the gap between state-of-the-art natural language processing techniques and real-world clinical applications in suicide prevention, addressing the lack of explainable transformer predictions for suicide risk assessment using clinical textual inputs validated for clinical utility. We develop and test a model for real-time crises within crisis text lines, integrating transformer-based models with SHAPs and incorporating clinical utility metrics.

We hypothesize that our transformer-based model will outperform the traditional baseline model in predicting SI and advanced suicidal engagement (ASE). Additionally, the SHAPs application is expected to highlight interpretable language features influencing the classification process, providing clinically relevant insights for decision-making in suicide prevention. This research aims to advance both the technical capabilities and practical applicability of ML in mental health assessment, potentially enhancing early intervention strategies in suicide prevention.

## Methods

### Overview

This study used a comprehensive approach to develop and evaluate ML models for identifying SI and behaviors in crisis helpline conversations. Our methodology encompassed several vital components, including data acquisition and preparation, model development and training, model evaluation and comparison, and explainability analysis. The statistical analysis comprised three main stages: preliminary sample analysis, development and training of two neural networks, and comprehensive evaluation of model performance. The following sections detail each component of our methodology, including data collection, preprocessing, model architecture, training procedures, and evaluation metrics.

### Study Design

**Figure 1:**
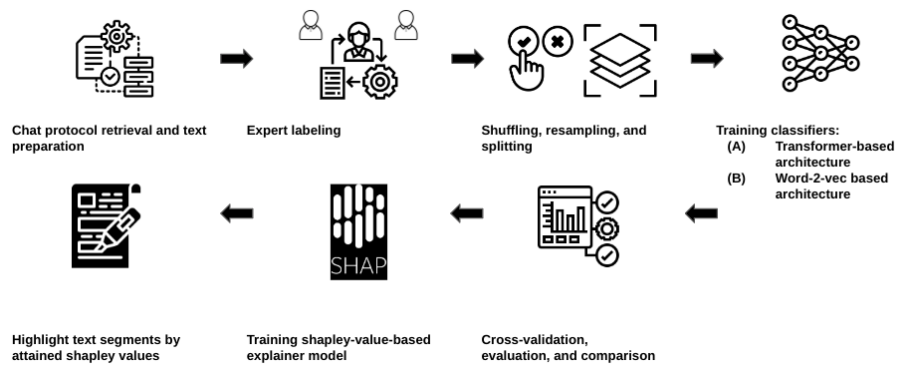
Study design for suicidality prediction model development and evaluation using German youth crisis helpline session transcripts collected between November 11, 2021, and April 30, 2022: data flow, model development, and evaluation process for image sources.

This study consists of several steps (Figure 1 [65-71]). We obtained chat protocols from krisenchat, a German crisis text line for youth. The dataset underwent cleaning, preprocessing, and expert labeling.

Post labeling, the data were randomized, partitioned, resampled, and encoded for model training. We then trained two text classification models and assessed model performance using various metrics. Lastly, we used a SHAPs explainer model to interpret the classification results.

### Data Collection and Preprocessing

We obtained chat transcripts from krisenchat, a prominent German preclinical crisis intervention service for individuals up to 25 years of age [72]. The dataset comprised sessions conducted between November 30, 2021, and April 30, 2022. We excluded sessions from individuals younger than 14 years of age and those lacking age information due to informed consent considerations. The final dataset included transcripts from individuals experiencing suicidal thoughts and behaviors, as well as those seeking advice on other topics.

We defined a chat session as all messages from a single individual during a counseling session, typically at most 2 hours. We removed all personal identifiers and preprocessed the text data to enhance readability and reduce variability. This process eliminated extraneous elements such as links, HTML tags, and special characters beyond typical German punctuation.

From sessions marked with SI by krisenchat counselors, we identified relevant conversations addressing SI within this study’s period. We excluded sessions too brief to assess suicidal ideation and behaviors (SIBs) or those terminating prematurely. Of 14,073 sessions, we identified 3193 control sessions, with 2886 meeting length and age criteria. We randomly selected 500 nonsuicidal sessions for manual review, replacing any indicating suicidal thoughts or severe self-harm with nonsuicidal cases. We used the imbalanced-learn library to address the class imbalance in our dataset by applying random oversampling of minority classes in the test set after splitting.

### Measures and Labeling

We used the Columbia-Suicide Severity Rating Scale (C-SSRS), the gold standard for assessing SIB [73,74]. The C-SSRS categorizes ideation levels from a general desire to die to actual suicide attempts, quantifying severity and intensity. It defines suicidal behavior as any life-ending action, including nonharmful behaviors, interrupted attempts, and preparatory actions.

To address data imbalance and align with recent suicidology theories [75,76], we applied composite categories of SI and ASE based on C-SSRS levels. This approach reflects the distinct processes of SI development and progression to suicidal behaviors [77]. The probability of acting on suicidal thoughts is influenced by an individual’s capacity to endure pain and access to lethal means [78].

We defined SI using C-SSRS items “wish to be dead” and “nonspecific active suicidal thoughts,” encompassing thoughts from the desire not to wake up to explicit life-ending statements. ASE comprised C-SSRS items involving specific methods, intent, or attempts, including contemplation of lethal methods, setting definite dates for action, and engaging in preparatory behaviors. The consideration of lethal means marks the transition to suicidal behaviors, defining the ASE category.

Three independent expert raters, extensively trained in C-SSRS, labeled the sessions. Their training involved mutual panel ratings and group discussions across 50 trial conversations not included in this study. Each rater subsequently assigned ratings independently. Raters conducted integrity discussions between rating sessions to maintain consistency and mitigate observer drift, exchanging thoughts and rating standards. Upon completing all session ratings, the raters convened to address discrepancies and reach a consensus on final ratings through comprehensive discussions.

### Reporting Standards

This study adheres to the Transparent Reporting of a Multivariable Prediction Model for Individual Prognosis or Diagnosis for artificial intelligence (AI) abstract and reporting guidelines checklist for prediction model development and validation [79] (Tables S1 and S2 in Multimedia Appendix 1) and the CONSORT-AI (Consolidated Standards of Reporting Trials for Artificial Intelligence) checklist [80] (Table S3 in Multimedia Appendix 1).

### Ethical Considerations

This study was conducted using ethical standards for human participant research. This study’s protocol received approval from the Ethics Committee of the International Psychoanalytic University Berlin (2023_08). Informed consent was obtained through krisenchat’s terms of service, which explicitly state that user data may be used for research purposes without direct identification of individuals. All personally identifiable information was removed from the chat transcripts during data preprocessing to protect privacy and confidentiality. Participants were not compensated directly, as this study used existing data from the crisis helpline. No images of participants were used in this study; the source text was solely used for training the classifier and was cleaned of all personally identifiable information. These measures ensure that no identification of individual participants is possible.

### Software

All preprocessing, modeling, and evaluation tasks were performed using TensorFlow’s [81] official Docker Image to construct a containerized GPU runtime on a Linux machine, using Python (version 3.8.1; Python Software Foundation) and CUDA (version 11.4; NVIDIA Corp). The machine had a 4 GB GPU and 16 GB of RAM. We used NumPy [82] and pandas [83] for preprocessing, while the Imblearn library was used for resampling. Model architectures and weights were sourced from the Hugging Face Transformers [35] library, and we used TensorFlow’s Keras [84] module for modeling. Visualization and metrics were facilitated through matplotlib [85], seaborn [86], sci-kit-learn [87], and sklearn [88]. The final script is openly accessible on GitHub [89].

### Model Architecture

#### Overview

One-way ANOVA to evaluate differences in word usage and age across 3 groups: nonsuicidal, SI, and ASE.

We developed two neural network models to classify SI and ASE in crisis interactions. Both models share a similar architecture, comprising three main blocks: an embedding block for the mathematical representation of messages, a long short-term memory (LSTM) block to model temporal dependencies, and a classification block.

Model 1, a transformer-multilayer perceptron (T-MLP), uses a pretrained transformer encoder (XLM-RoBERTa-base) [90], available on Hugging Face [35]. This multilingual model, trained on 2.5 TB of CommonCrawl data in 100 languages, tokenizes and encodes input text into a 768-dimensional embedding. Each message is embedded separately and then attached to an array of embedded messages. The embeddings feed into a time-distributed dense layer, followed by an LSTM encoder with dropout regularization, and finally, a multilayer perceptron for classification.

Model 2, a word2vector-multilayer perceptron (W2V-MLP), uses pretrained German word-vector embeddings (300-dimensional) processed through convolutional filters. These word-wise embeddings, trained using FastText on standard crawl and Wikipedia datasets, form an embedding matrix. This matrix is processed through a convolutional layer, max pooling, dropout, an LSTM encoder, and a multilayer perceptron layer for classification.

Both models output probabilities for three categories: not suicidal, SI, and ASE. Detailed model parameters are provided in Table S1 in Multimedia Appendix 2.

#### Model Training, Cross-Validation, and Evaluation

We implemented a three-phase evaluation and cross-validation protocol: (1) cross-validation of training, (2) post hoc estimation of overall evaluation metrics, and (3) assessment of calibration and clinical utility metrics.

To evaluate model stability, we performed 5 iterations of repeated cross-validation. We initialized data at random seeds, then shuffled and partitioned it (1011 for training and 337 for testing), maintaining consistent class ratios through stratified shuffling. We applied random oversampling to both sets individually to ensure balanced class ratios.

We limited training to a maximum of 100 epochs, incorporating an early stopping mechanism triggered by test accuracy, with a patience setting of 5 epochs. We selected the best model based on its area under the curve (AUC) performance, derived from receiver operating characteristics (ROCs).

We assessed model performance using standard class-wise metrics, including AUC, precision, recall, and *F*_1_-scores, computing these metrics macroaveraged across classes. We determined class-wise metrics using a one-versus-all approach, binarizing predictions for each class. AUC measures the model’s discriminative ability, with scores of 0.7-0.8 considered acceptable and 0.9 indicating high discrimination. We computed ROC curves using the one-versus-all method, quantifying the model’s classification ability across all thresholds [80]. We applied bootstrapping with 1000 subsamples from the test dataset to establish 95% CIs [81]. We provided confusion matrices to visualize misclassifications [82].

To demonstrate clinical utility, we conducted decision curve analysis (DCA) for the SI and ASE classes [61,62]. DCA calculates a “net benefit” (NB) value based on the harm-to-benefit ratio of clinical decisions. An NB of 0.2 indicates the model detects 20 true positives without increasing false positives. We set threshold probabilities for SI between 0.3 and 0.5 and for ASE between 0.15 and 0.30 based on our sample’s event rates and clinical considerations [63].

We assessed model calibration by calculating the overall Brier score (BS), which measures the mean square error of prediction probabilities against actual outcomes. Lower BS values indicate better calibration accuracy. We decomposed the BS into reliability, resolution, and uncertainty to offer insights beyond AUC-ROC [61]. We also calculated the Brier skill score to compare performance improvements of our transformer model over the word2vec model in calibration.

We visually assessed calibration using plots depicting the relationship between predicted probabilities and observed outcome frequencies [86]. A perfectly calibrated model aligns with the plot’s diagonal; deviations indicate under- or overprediction.

Detailed DCA calculations, rationale, interpretations, and BS decomposition analysis are available in Table S3 in Multimedia Appendix 2.

### Explainability Analysis

We used a SHAPs model-agnostic post hoc explainer to enhance our understanding of the models’ prediction processes. Shapley values, derived from cooperative game theory [91], provide local explanations for each prediction [92]. These values quantify each feature’s impact on an instance’s prediction relative to its average impact across other feature combinations. The algorithm uses tokenized text vectors as inputs and considers all possible feature combinations to calculate these contributions.

In addition to examining individual results, we determined the overall importance of specific language features in predicting nonsuicidal, SI, or ASE categories. This approach provides a comprehensive understanding of the linguistic markers that influence the model’s decision-making process.

## Results

### Overview

The final dataset comprised 1348 eligible sessions, partitioned into 1011 training and 337 testing cases. After resampling, the training set expanded to 1254 cases, and the test set to 420 cases. We provide dataset characteristics in Table S2 in Multimedia Appendix 2. The mean participant age was 17.9 (95% CI 17.7-18.1) years. We observed significant differences in word count across groups (*F*_2,97_=48.34, *P*<.001).

### Model Training, Evaluation, and Cross-Validation and Evaluation

The training curves demonstrated that the transformer model learned consistently and stably (Figure S1 in Multimedia Appendix 3). In contrast, the baseline word vector model rapidly reached its learning peak before exhibiting overfitting, halted only by the early stopping mechanism (Figure S1 in Multimedia Appendix 3). The Training Curves section in Multimedia Appendix 3 provides a more detailed interpretation of these learning curves.

The transformer model (T-MLP) achieved superior performance with an overall accuracy of 0.79 (95% CI 0.73-0.99) and a macroaveraged AUC-ROC of 0.89 (95% CI 0.81-0.91). It demonstrated excellent discrimination for nonsuicidal sessions (AUC-ROC=0.96, 95% CI 0.96-0.99) and good discrimination for SI and ASE with AUC-ROCs of 0.85 (95% CI 0.97-0.86) and 0.87 (95% CI 0.81-0.88), respectively. The baseline word2vec model (W2V-MLP) showed lower performance across all metrics (Table 1).

**Table 1:** Classification performance metrics of transformer-based (T-MLP^a^) and word vector-based (W2V-MLP^b^) models for predicting NS^c^, SI^d^, and ASE^e^ in German youth crisis helpline users (N=337 evaluation set).

Model and class			Precision (positive predictive value)		Recall (sensitivity)		*F*_1_-score (harmonic mean of precision and recall)		AUC^f^-ROC^g^ (95% CI)
**T-MLP**									0.89 (0.81-0.91)
	NS	0.97		1		0.98		0.96 (0.96-0.99)	
	SI	0.71		0.65		0.68		0.85 (0.97-0.86)	
	ASE	0.69		0.72		0.71		0.87 (0.81-0.88)	
**W2V-MLP**									0.77 (0.64-0.9)
	NS	0.61		0.86		0.71		0.89 (0.84-0.9)	
	SI	0.59		0.69		0.61		0.78 (0.68-0.78)	
	ASE	0.67		0.28		0.39		0.64 (0.62-0.72)	

^a^T-MLP: transformer-multilayer perceptron.

^b^W2V-MLP: word2vector-multilayer perceptron.

^c^NS: not suicidal.

^d^SI: suicidal ideation.

^e^ASE: advanced suicidal engagement.

^f^AUC: area under the curve.

^g^ROC: receiver operating characteristic.

### Evaluation and Cross-Validation

**Figure 2:**
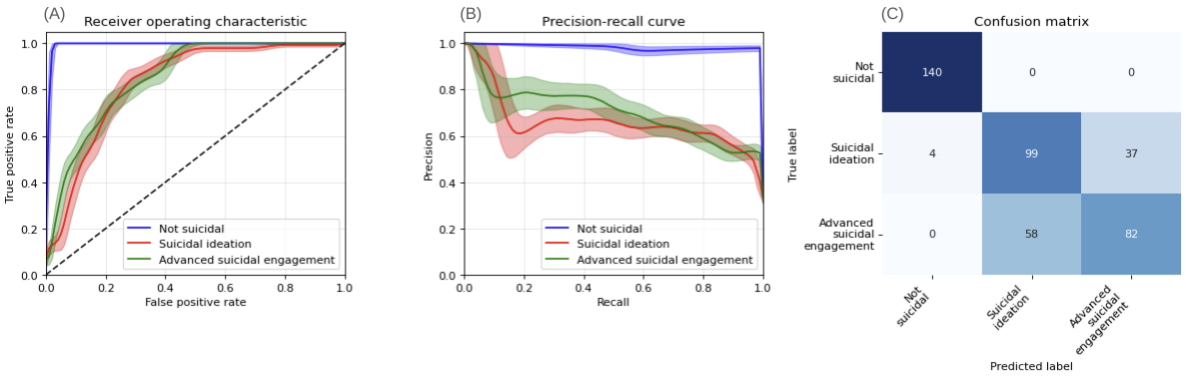
Performance analysis of the transformer-based model (T-MLP) for predicting not suicidal, SI, and ASE based on session transcripts of German youth crisis helpline users between November 30, 2021, and April 30, 2022 (N=337 evaluation set). (A) Class-wise one-versus-all AUC-ROC analysis with 95% CIs derived from 1000 bootstrap samples. (B) Class-wise precision-recall analysis with 95% CIs derived from 1000 bootstrap samples. (C) Confusion matrix of the test set: light colors represent low numbers, dark colors represent high numbers; correct classifications are on the diagonal from top-left to bottom-right. ASE: advanced suicidal engagement; AUC: area under the curve; SI: suicidal ideation; ROC: receiver operating characteristic; T-MLP: transformer-multilayer perceptron.

Class-wise metrics demonstrate the transformer model’s consistent superiority over the baseline model across all classification metrics. Figure 2 illustrates the performance of the T-MLP model. The AUC-ROC analysis (Figure 2A) confirms the model’s high discriminative ability across different classes and threshold probabilities. Precision-recall curves (Figure 2B) reveal some uncertainty at lower recall thresholds, suggesting potential for improved calibration. The confusion matrix (Figure 2C) demonstrates the model’s effectiveness in distinguishing nonsuicidal cases while showing some inconsistency in classifying SI and ASE.

**Figure 3:**
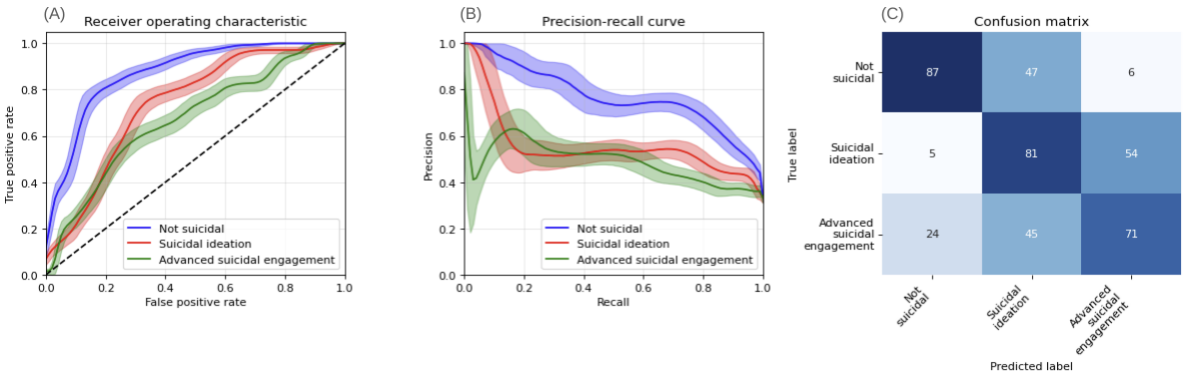
Performance analysis of word vector-based model (W2V-MLP) for predicting not suicidal, SI, and ASE in German youth crisis helpline users (N=337 evaluation set). (A) Class-wise one-versus-all AUC-ROC analysis with 95% CIs derived from 1000 bootstrap samples. (B) Class-wise sensitivity-specificity analysis with 95% CIs derived from 1000 bootstrap samples. (C) Confusion matrix of the test set: light colors represent low numbers, dark colors represent high numbers; correct classifications are on the diagonal from top-left to bottom-right. ASE: advanced suicidal engagement; AUC: area under the curve; SI: suicidal ideation; ROC: receiver operating characteristic; W2V-MLP: word2vector-multilayer perceptron.

The T-MLP model exhibits a low false negative rate of 0.01, indicating high accuracy in identifying suicidal sessions. Conversely, the W2V-MLP model (Figure 3) demonstrates inferior performance. Its AUC-ROC analysis (Figure 3A) and precision-recall curves (Figure 3B) show lower discriminative ability. The confusion matrix (Figure 3C) reveals a tendency to over-predict ASE, with a higher false negative rate of 0.17 and a false positive rate of 25% for nonsuicidal outcomes.

These results underscore the T-MLP model’s enhanced capability in accurately classifying SIBs compared to the baseline W2V-MLP model while also highlighting areas for potential improvement in model calibration.

**Figure 4:**
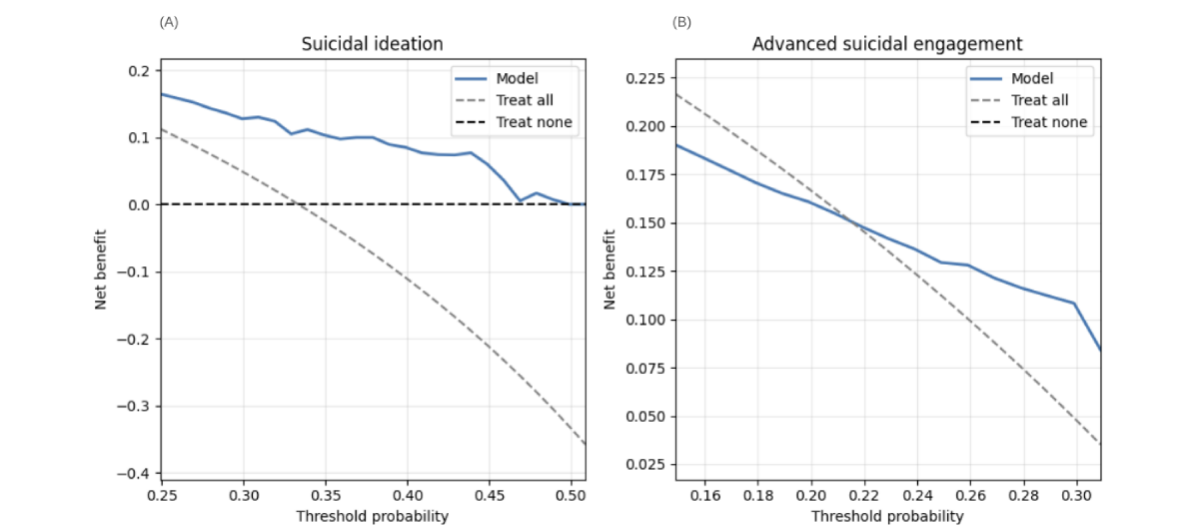
Decision curve analysis of the transformer-based model (T-MLP) for predicting suicidal ideation and advanced suicidal engagement in German youth crisis helpline users (N=337 evaluation set). (A) Class-wise decision curve analysis for suicidal ideation. (B) Class-wise decision curve analysis for advanced suicidal engagement. Color coding: treat-all strategy in gray, treat-none strategy in black, classifier (T-MLP) in blue, theoretical maximal benefit in red. Irrelevant threshold segments are grayed out. T-MLP: transformer-multilayer perceptron.

**Figure 5:**
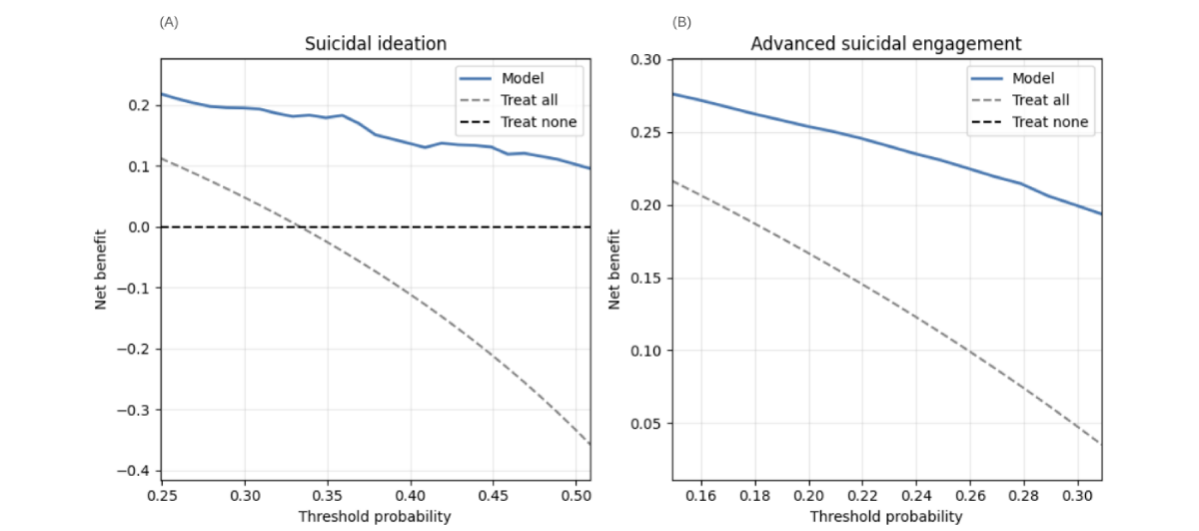
Decision curve analysis of word-vector model (T-MLP) for predicting suicidal ideation and advanced suicidal engagement in German youth crisis helpline users (N=337 evaluation set). (A) Class-wise decision curve analysis for advanced suicidal engagement. (B) Class-wise decision curve analysis for advanced suicidal engagement. Color coding: treat-all strategy in gray, treat-none strategy in black, classifier (T-MLP) in blue, theoretical maximal benefit in red. Irrelevant threshold segments are grayed out. T-MLP: transformer-multilayer perceptron.

DCA demonstrates the T-MLP model’s superior clinical utility. For SI prediction, the T-MLP model yields higher NBs than treat-all and treat-none strategies within 0.3-0.5 threshold probabilities, identifying 10-18 true positives without increasing false positives (Figure 4A). The W2V-MLP model, while initially beneficial, exhibits potentially detrimental clinical decisions beyond 0.4 thresholds (Figure 5A).

For ASE, the T-MLP model outperforms alternative strategies within 0.15-0.3 thresholds, achieving an NB of 0.20-0.25 (Figure 4B). Conversely, the W2V-MLP model underperforms relative to the treat-all strategy, potentially leading to suboptimal clinical decisions (Figure 5B). These findings underscore the T-MLP model’s enhanced clinical applicability in suicide risk assessment.

**Figure 6:**
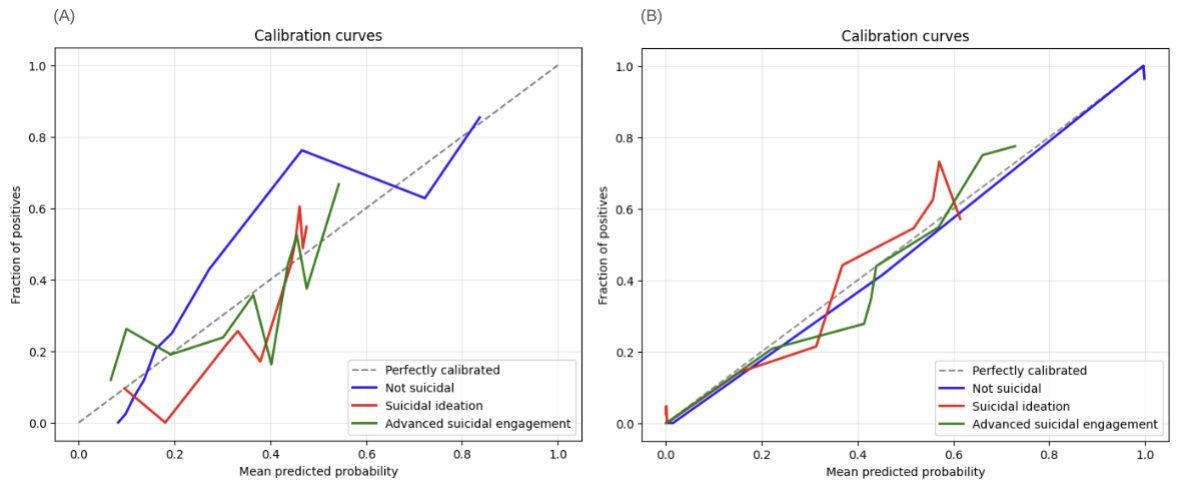
Calibration analysis of transformer-based (T-MLP) and word vector-based (W2V-MLP) models for predicting nonsuicidal, suicidal ideation, and advanced suicidal engagement based on session transcripts of German youth crisis helpline users between November 30, 2021, and April 30, 2022 (N=337 evaluation set). (A) Class-wise calibration curves for the T-MLP model. (B) Class-wise calibration curves for the W2V-MLP model. Color coding: not suicidal (blue), suicidal ideation (orange), and advanced suicidal engagement (green). T-MLP: transformer-multilayer perceptron; W2V-MLP: word2vector-multilayer perceptron.

We assessed model calibration using Brier and Log loss scores. The T-MLP model achieved lower scores (Brier: 0.10, Log loss: 0.50) compared to the W2V-MLP model (Brier: 0.18, Log loss: 0.92), indicating superior calibration performance. However, both models exhibited resolution challenges in specific classes (Table 2, and Figures 5 and 6).

The Brier Skill Score revealed a 44.4% improvement in classification performance by the T-MLP model over the baseline. Both models demonstrated low variability in predicted probabilities across samples, with an overall uncertainty of 0.22 due to class rebalancing.

Calibration curve analysis (Figure 6) exposed distinct prediction patterns. The T-MLP model consistently overpredicted for the nonsuicidal class across all decision thresholds (Figure 6A) while underpredicting SI and ASE at probabilities above 0.5 and overpredicting at lower thresholds. The W2V-MLP model underpredicted for the nonsuicidal class up to a 0.6 threshold and overpredicted suicidal outcomes at lower thresholds (Figure 6B).

These calibration patterns suggest potential overestimation of clinical utility within predefined thresholds for both models, necessitating cautious interpretation of the DCA results.

**Table 2:** Brier score decomposition and model comparison for transformer-based (T-MLP^a^) and word vector-based (W2V-MLP^b^) models in predicting NS^c^, SI^d^, and ASE^e^ based on session transcripts of German youth crisis helpline users between November 30, 2021, and April 30, 2022 (N=337 evaluation set).

	Transformer^f^			word2vec^g^		
Class	NS	SI	ASE	NS	SI	ASE
Reliability	0.002	0.007	0.008	0.001	0.010	0.011
Resolution	0.219	0.863	0.106	0.06	0.015	0.023
Uncertainty	0.222	0.222	0.222	0.222	0.222	0.222

^a^T-MLP: transformer-multilayer perceptron.

^b^W2V-MLP: word2vector-multilayer perceptron.

^c^NS: not suicidal

^d^SIS: suicidal ideation.

^e^ASE: advanced suicidal engagement.

^f^Average Brier loss=0.10; Brier skill score=44.4%.

^g^Average Brier loss=0.18; Brier skill score=44.4%.

### Explainability Analysis

We implemented SHAPs to generate additive text explanations for the transformer model. Due to privacy constraints, we present a vignette illustrating the model’s interpretation of feature importance rather than specific outputs.

Global feature importance analysis revealed linguistic traits associated with SI, including self-reference, negation, expressions of low self-esteem, problem emphasis, and articulations of isolation. ASE texts exhibited similar characteristics with additional absolutist language and frequent references to lethal methods and injuries.

**Figure 7:**
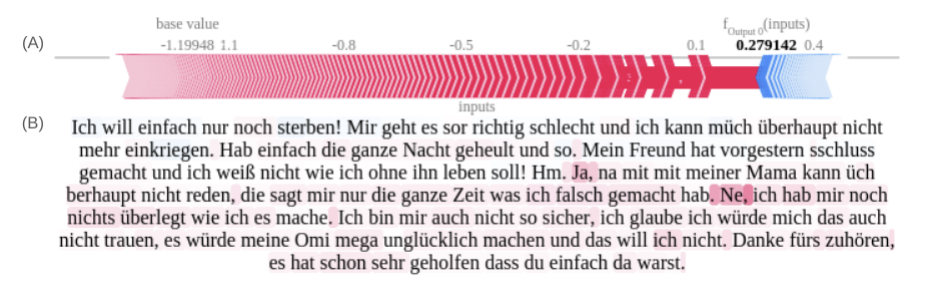
Shapley values-based text explanation plot for nonsuicidal outcome prediction in German youth crisis helpline users, plot based on synthetic case vignette. (A) Force plot of aggregated Shapley values indicating the predominance of not suicidal features over SI-associated risk markers. (B) The text highlights illustrating content associated with the target class (not suicidal) in red and suicidal content in blue. SI: suicidal ideation.

**Figure 8:**
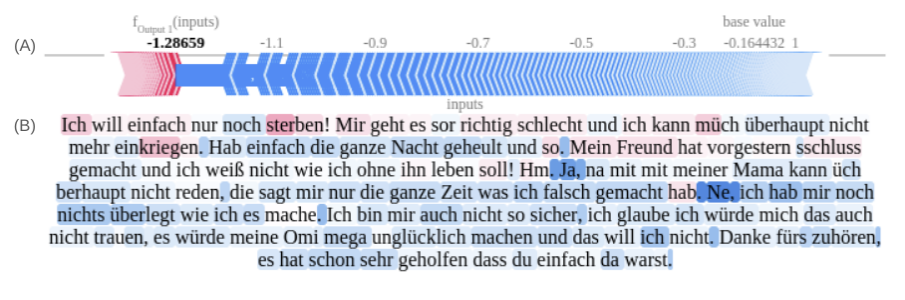
Shapley values-based text explanation plot for suicidal ideation outcome prediction in German youth crisis helpline users, plot based on synthetic case vignette. (A) Force plot of aggregated Shapley values indicating the balance between suicidal ideation-associated risk markers and nonsuicidal features. (B) Text highlights illustrating content associated with the target class (suicidal ideation) in red and nonsuicidal content in blue.

**Figure 9:**
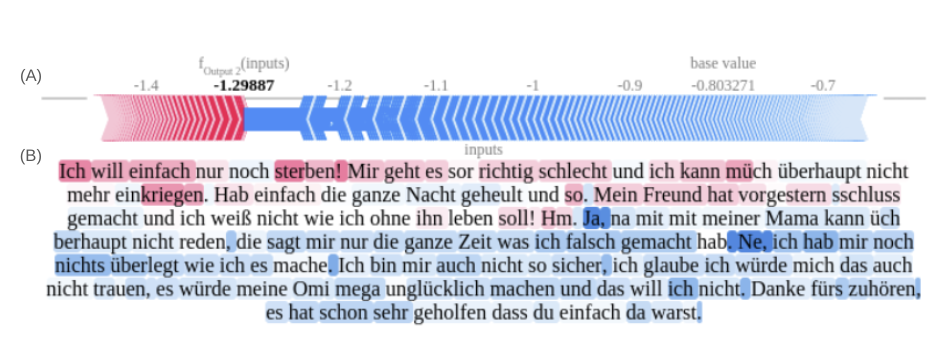
Shapley values-based text explanation plot for advanced suicidal engagement outcome prediction in German youth crisis helpline users, plot based on synthetic case vignette. (A) Force plot of aggregated Shapley values indicating the balance between advanced suicidal engagement–associated risk markers and nonsuicidal features. (B) Text highlights illustrate content associated with the target class (advanced suicidal engagement) in red and nonsuicidal content in blue. Translation: “I just want to die right now. I am feeling pretty bad, and I can’t calm myself down. I was crying the whole night and stuff. My boyfriend dumped me the day before yesterday, and I don't know how to live without him!” “Hm. Yeah, I can't talk to my mum about such stuff. She just points out my flaws all the time.” “No, I don't have a specific Plan of how to do it; I am also pretty unsure - I think I would never have the courage to do it, my Granny would be super sad and I don't want that. Thank you for listening to me. Thank you for being there for me. This helped a lot.”.

Figures 7A and 7B depict a synthetic session vignette predicting the nonsuicidal group, highlighting de-escalating statements in the latter half of the conversation as influential for a positive prediction. Figures 8A, 8B, 9A, and 9B illustrate tokens associated with SI and ASE, respectively. These figures emphasize self-referential statements, expressions indicating a desire to end one’s life, and words associated with negative sentiment.

It is noteworthy that in transformer architectures, word tokens do not have fixed impacts on the output due to their context-dependent evaluation. This contextual approach, while representing an advancement over other word-based methods, limits the generalizability of derived Shapley values and plots. Interestingly, the model did not assign high importance to the misspelled word “sschluss gemacht” (intended as “schluss gemacht” meaning “broke up”), demonstrating its robustness to typographical errors.

## Discussion

### Overview

We aimed to develop, evaluate, cross-validate, and benchmark a transformer-based prediction model for detecting SI and ASE among adolescents seeking emergency online counseling services in Germany.

The primary findings of this study demonstrate the proficiency of our transformer-based prediction model in identifying SI and ASE among adolescents using online counseling services. Our model outperformed the baseline word-vector approach, exhibiting excellent to very good performance in distinguishing between nonsuicidal sessions, SI, and ASE. Additionally, the Shapley analysis successfully identified word tokens associated with suicidal outcomes, providing valuable insights into language features indicative of SI. These findings highlight the potential of ML models in enhancing suicide prevention efforts.

### Principal Results

Our key findings underscore the superior performance of our transformer-based prediction model in identifying SI and ASE using natural language data from adolescents. The model was trained on rigorously collected data evaluated by 3 expert raters using an established and validated scale. We adhered to a strict validation protocol, including repeated cross-validation and clinical utility metrics, to ensure our results are reliable, interpretable, and applicable in clinical settings.

The transformer model showed strong capabilities in differentiating between session transcripts containing nonsuicidal statements and those featuring discussions of SI or ASE, despite some calibration challenges. These findings suggest the model’s potential as a valuable screening tool for identifying individuals who may be at risk for SI and ASE, potentially supporting clinical decision-making and enabling timely interventions. The model excelled at recognizing transcripts with nonsuicidal content, though it faced some difficulties in precisely distinguishing more nuanced levels of SIB. Notably, our model substantially surpassed the performance of the baseline model that used pretrained word vectors, demonstrating improved overall metrics and a reduced risk of misclassification. While the model achieved high sensitivity and specificity in identifying nonsuicidal cases, its specificity for SI and ASE was comparatively lower.

Analysis of calibration curves indicates a necessity for recalibration before clinical implementation, as current reliability metrics suggest some degree of instability. Although the transformer model exhibits evident clinical utility, the observed calibration issues may lead to an overestimation of its practical performance. Furthermore, while the application of SHAPs analysis effectively identified word tokens associated with suicidal outcomes, it demonstrated reduced effectiveness in interpreting out-of-context tokens. This limitation potentially constrains the model’s capacity to comprehensively capture the dynamic nature of youth language patterns.

Overall, the transformer model outperformed the word-vector model, demonstrating superior performance and higher clinical utility, even when trained on a relatively small, domain-specific dataset. This advantage primarily stems from the transformer encoders’ advanced ability to represent natural language, partly due to their contextual embedding strategy and training on larger datasets. Additionally, the architecture inherently excels at analyzing deeper levels of language structure.

Our findings offer strong support for the use of ML models, particularly those using transformers and transfer learning, in detecting and classifying levels of SI in clinical psychological and psychiatric research that relies heavily on text and speech data. The transformer model surpasses both the treat-all and treat-none strategies and the baseline model for detecting SI and ASE. Including an explainability component provides clinicians with insights into the specific language features associated with SI or advanced engagement [93].

The SHAPs algorithm generated local explanations for the model’s predictions, which we aggregated to gain insight into the global importance of features in the language of suicidal adolescents. The explainer model effectively pinpointed specific language markers that influence the model’s predictions. The importance of explainability is underscored by regulations such as the AI Act of the European Union, which emphasizes transparency in AI applications [94].

The language markers identified by the model are consistent with existing research on suicidal language markers, validating our results. Notably, our analysis confirmed that the usage of first-person singular pronouns and absolutist language is linked to SI [95-97].

This study demonstrates the potential of our model to accurately assess and identify suicidal thoughts in adolescents, with significant implications for prevention strategies addressing SIBs. The model’s high applicability to the online crisis counseling context facilitates its potential adoption by other online counseling services, potentially aiding in risk mitigation and supporting clinical decision-making processes. The model’s capacity to identify key risk markers may enable more precise risk assessments and inform targeted interventions for individuals at elevated risk of ASE. Furthermore, the model’s ability to track and detect changes in language usage over time could contribute to earlier identification of individuals experiencing increased risk.

In summary, the developed model can significantly enhance the effectiveness and efficiency of prevention efforts at any level of SI.

### Limitations

The primary limitation of this study is the small sample size, which may restrict the generalizability of the model’s performance. As evidenced by the learning curves (Figures S1 and S2 in Multimedia Appendix 3), the transformer model converged with minimal overfitting in contrast to the baseline model. The limited sample size might have influenced class learnability, particularly for the word-vector approach, which requires more parameter estimation. Repeated cross-validation revealed training instability and sensitivity to weight initialization, potentially leading to class-specific biases. Calibration metrics indicated significant reliability issues for both models, suggesting the need for recalibration before clinical implementation. Due to these calibration concerns, the clinical utility of the models should be interpreted cautiously, as small sample sizes introduce more noise to neural networks [98]. These issues could be mitigated by using larger datasets, implementing robust weight initialization, or applying additional regularization techniques.

Furthermore, due to metric requirements, we applied resampling techniques to the rarer outcomes, which may lead to overfitting minority classes and lower generalization. Performance improvements over the baseline may be overstated due to less emphasis on feature engineering in the word-vector model. Incorporating n-grams, tf-idf (term frequency–inverse document frequency), or dictionary-based features could address the lack of sequential text structure in word embeddings, which this study did not fully address.

Potential age or gender biases in the expression of suicidality may exist, as the sample was not gender-balanced, potentially skewing results toward female expressions of SIBs. The class imbalance necessitated the use of oversampling techniques, which might introduce biases. Technical limitations led to session truncations, affecting data quality and representativeness.

This study’s exclusive focus on textual data, while appropriate for the crisis text line setting, may limit the comprehensive assessment of suicidality. Suicidal behavior is multifaceted, and other data types such as audio or visual cues could provide additional valuable information. The absence of these modalities in our analysis is a limitation inherent to the text-based nature of the crisis line service studied.

Token-based post hoc explanations might not fully capture the sequential nature of speech, possibly missing complex effects detected by transformers and LSTM layers. Future research should explore specific techniques for transformer-based explainability and evaluate the linguistic features of highlighted tokens. Limiting analysis to text tokens may overlook the importance of other communication forms, such as emoticons, which play a significant role in conveying meaning. Future models should consider these aspects.

Additionally, the vignette demonstrated that despite being trained on more extensive data, the transformer encoder still will not fully capture irregular or misspelled language. Adapting to new language variations, often developed among youth, heavily depends on collecting domain-specific datasets for embedding models.

### Comparison to Prior Work

The discriminative abilities of our model are comparable, albeit slightly lower, to those reported by Zhang et al [99], who analyzed 659 suicide notes, 431 last statements, and 2000 neutral posts. Their model achieved 95%, 94.9%, and 94.9% in precision, recall, and *F*_1_-score, respectively, although it was limited to binary targets and explicitly focused on suicide notes. Similarly, Broadbent et al [28] found that using sentence-wise embeddings of counseling sessions, a transformer embedding surpassed a baseline tf-idf model in reducing the false-negative rate.

In contrast, Aldhyani et al [100] demonstrated that a convolutional neural network and bidirectional LSTM model excelled over the XGBoost model, achieving 95% accuracy in detecting SI compared to the latter’s 91.5%. However, when using validated dictionary features such as Linguistic Inquiry and Word Count, boosted tree algorithms performed better than a neural network architecture combining convolutional and LSTM layers (convolutional neural network and bidirectional LSTM) on binary targets. The key distinction of this study from Aldhyani et al [100] lies in the emphasis on feature engineering and the use of dictionaries, which were leading methods for building text classifiers before the emergence of transfer learning with large pretrained language models [62].

Unique to our study, we also include metrics for calibration and clinical utility, enhancing the practical value of our findings.

### Generalizability

The generalizability of our findings varies by domain. Given the small and domain-specific sample, the results may primarily apply to German-speaking adolescents seeking mental health assistance. However, the analysis of language features suggests that findings might extend to a broader demographic group.

### Clinical Implications and Implications for Future Research

Our transformer-based model has the potential to assist clinicians in identifying at-risk individuals and improving intervention prioritization. Continuous monitoring using such models is cost-effective and can detect cases that might otherwise be missed, facilitating referral to qualified human review and leading to more accurate and timely interventions. This research significantly contributes to prevention efforts addressing SIBs.

Despite the promise of such models, as indicated in our study, future research could enhance the model’s accuracy and utility. Subsequent studies should aim to collect sample sizes that are adequate for the power requirements of ML. If faced with the common problem of class imbalance, researchers could opt for adjusting the cost function of the learner or using synthetic data generation through augmentation to mitigate this issue.

Other key priorities include validating the model across diverse clinical settings such as inpatient facilities, rural clinics, and school-based mental health services to enhance its generalizability. Additionally, evaluating the model’s performance across different demographic groups and cultural contexts will be essential to ensure its effectiveness and identify potential biases.

Developing dynamic models that track changes over time and incorporate additional factors such as demographics could improve performance. This could be achieved by using retrained transformers in extracting longitudinal event data [101] or by incorporating explanations into the clinicians’ counseling environment [102]. Integrating transformer embeddings into multimodal graph network models might refine precision and bolster prevention efforts by addressing the multifactorial nature of suicide.

Integrating multiple data types could provide a more holistic view of an individual’s mental state. However, such approaches would require careful consideration of privacy concerns, data integration challenges, and the development of more complex models capable of processing diverse input types. Future studies could explore the relative contributions of different data modalities to suicide risk assessment and the feasibility of implementing multimodal systems in various clinical settings.

ML shows potential in mental health applications, but it should be considered a complementary tool to clinical expertise and evidence-based decision-making rather than a standalone solution. In suicidology, it is imperative to recognize that suicide risk emerges from complex life circumstances that require comprehensive social interventions, support systems, and targeted public policies. While ML algorithms can serve as diagnostic tools to detect linguistic markers associated with suicide risk in individuals, they cannot independently prevent or resolve the underlying personal challenges. Furthermore, the implementation of ML in this context necessitates careful ethical considerations to ensure that these tools augment human judgment without introducing or amplifying biases. Future research should focus on integrating ML approaches within broader, multifaceted suicide prevention strategies that address the full spectrum of risk factors and protective measures.

### Conclusions

Suicide prevention represents a critical public health concern, particularly among adolescents. Timely identification of individuals displaying SIBs is crucial for early intervention. Our study introduces an explainable transformer-based ML model that outperforms a baseline word-vector approach in identifying SI and ASE in adolescents seeking help via a German crisis helpline. This model could be invaluable for clinicians prioritizing intervention cases. However, challenges remain in discerning finer-grained types of SI. This research underscores the potential of ML in detecting SI and represents a step toward more effective suicide prevention. Future work should focus on enhancing model accuracy in longitudinal setups or incorporating multimodal features.

## Appendix

Multimedia Appendix 1 Reporting guideline checklists.

Multimedia Appendix 2 Supplementary tables and figures.

Multimedia Appendix 3 A primer on neural networks and metrics.

## Abbreviations

AI: artificial intelligence
ASE: advanced suicidal engagement
AUC: area under the curve
BS: Brier score
C-SSRS: Columbia-Suicide Severity Rating Scale
CONSORT-AI: Consolidated Standards of Reporting Trials for Artificial Intelligence
DCA: decision curve analysis
LSTM: long short-term memory
ML: machine learning
NB: net benefit
ROC: receiver operating characteristic
SHAP: Shapley Additive Explanation
SI: suicidal ideation
SIB: suicidal ideation and behavior
tf-idf: term frequency-inverse document frequency
T-MLP: transformer-multilayer perceptron
W2V-MLP: word2vector-multilayer perceptron

## Data Availability

This study used highly sensitive and confidential crisis helpline chat transcripts from vulnerable individuals, including minors. Due to the nature of these conversations, which frequently contain personal details and sensitive information regarding mental health and SI, the raw data cannot be made publicly available. This restriction is necessary to protect participant privacy and confidentiality and to comply with ethical guidelines and data protection regulations, including the General Data Protection Regulation. Even in an anonymized form, the risk of reidentification cannot be completely mitigated, thus precluding data sharing.
