# Supplementary material for: An Explainable Artificial Intelligence Text Classifier for Suicidality Prediction in Youth Crisis Text Line Users: Development and Validation Study
Source: JMIR Public Health Surveill. 2025 Jan 29;11:e63809. doi: 10.2196/63809 (PMC11822322; doi:10.2196/63809)
Supplement: Multimedia Appendix 1 [file publichealth_v11i1e63809_app1.docx]

## **Tripod+AI Checklists**

The following abstract checklist is part of the Tripod+AI checklist, which was first published by Collins and Colleagues [1]. Items are marked in black, and responses and citations are in blue.

**Table S1.** Tripod+AI abstracts checklist.

| Section and item | Checklist item |
| --- | --- |
| **Title** | |
| 1 | Identify the study as developing or evaluating the performance of a multivariable prediction model, the target population, and the outcome to be predicted  “to train a machine learning (ML) model, utilizing a transformer-based language model architecture”  “We set out to i) apply ML methods in predicting suicidal ideation and behaviors in a real-world crisis-helpline dataset, using transformer-based pretrained models as a building block ii) evaluate, cross-validate, and benchmark the model against traditional text classification approaches, and ” |
| **Background** | |
| 2 | Provide a brief explanation of the healthcare context and rationale for developing or evaluating the performance of all models  “Suicide is an important and pressing avenue for public health”  “We used chat protocols from youth seeking help from a German crisis helpline,”  “Such models may potentially support clinical decision-making in the context of suicide prevention services.” |
| **Objectives** | |
| 3 | Specify the study objectives, including whether the study describes model development, evaluation, or both  “We set out to i) develop and apply ML methods in predicting suicidal ideation and behaviors in a real-world crisis-helpline dataset, using transformer-based pretrained models as a building block ii) evaluate, cross-validate, and benchmark the model against traditional text classification approaches” |
| **Methods** | |
| 4 | Describe the sources of data  “we used chat protocols from youth seeking help from a German crisis helpline” |
| 5 | Describe the eligibility criteria and setting where the data were collected “We used chat protocols from youth, aged 14 to 25, seeking help from a German crisis helpline,” |
| 6 | Specify the outcome to be predicted by the model, including time horizon of predictions in case of prognostic models “We predicted Suicidal Ideation (SI) and Advanced Suicidal Engagement(ASE), indicated by composite Columbia-Suicide-Severity-Rating Scale (C-SSRS) scores” |
| 7 | Specify the type of model, a summary of the model-building steps, and the method for internal validation† |
| 8 | Specify the measures used to assess model performance (eg, discrimination, calibration, clinical utility)  “We then obtained discrimination, calibration, clinical utility and explainability information using a Shapley value-based post-hoc estimation (SHAP) model.  ” |
| **Results** | |
| 9 | Report the number of participants and outcome events  “Based on data from 1,348 help-seeking encounters” |
| 10 | Summarise the predictors in the final model†  N.A for Neural network, but Explainability Information highlights ceratin features: “The SHAP model highlighted language features like 'I-talk,' phrases indicating low self-esteem and self-hatred, lethal means, hopelessness, and body issues as predictive of suicidal ideation and behaviors” |
| 11 | Report model performance estimates (with confidence intervals)  “the transformer-based classifier yielded a macro-averaged area under the curve (AUC) of 0.93 (95% CI [0.87, 0.99]) and a macro-averaged F1 score of 0.79 (95% confidence interval [CI] [0.60, 0.96]). It outperformed the word-vector-based baseline model (AUC = 0.77; 95% CI [0.63, 0.89]; F1 score = 0.56; 95% CI [0.0, 0.65])” |
| **Discussion** | |
| 12 | Give an overall interpretation of the main results  “Neural Networks, using LM-based transfer learning, can effectively identify suicidal ideation and advanced suicidal engagement.” |
| **Registration** | |
| 13 | Give the registration number and name of the registry or repository  did not register the study |

**Table S2.** Tripod+AI reporting guidelines checklist.

| Section/topic | Item | Development/  evaluation* | Checklist item |
| --- | --- | --- | --- |
| **Title** | | | |
| Title | 1 | D;E | Identify the study as developing or evaluating the performance of a multivariable prediction model, the target population, and the outcome to be predicted  “Development and Validation of an Explainable Transformer-Based Artificial Intelligence” |
| **Abstract** | | | |
| Abstract | 2 | D;E | See TRIPOD+AI for Abstracts checklist |
| **Introduction** | | | |
| Background | 3a | D;E | Explain the healthcare context (including whether diagnostic or prognostic) and rationale for developing or evaluating the prediction model, including references to existing models  “Accurate and timely identification of individuals showcasing suicidal ideation and behaviors, thus poses a significant challenge to mental health care and educational institutions working with youth.”  “However, the emergence of specifically text-based services has opened up new avenues for Machine Learning (ML) based prediction models in suicide prevention” |
|  | 3b | D;E | Describe the target population and the intended purpose of the prediction model in the context of the care pathway, including its intended users (eg, healthcare professionals, patients, public)  “to mental health care and educational institutions working with youth.”  “Using such models to accurately detect suicidal ideation and behaviors as well as to predict its degree of escalation can facilitate efforts to identify subjects at risk and support counselors in making clinical decisions regarding high-risk patients and time-critical intervention scenarios [2].” |
|  | 3c | D;E | Describe any known health inequalities between sociodemographic groups |
| Objectives | 4 | D;E | Specify the study objectives, including whether the study describes the development or validation of a prediction model (or both)  “We aimed to develop, evaluate, and compare state-of-the-art transformer-based classification models to predict relevant and pressing public health concern of suicidal ideation and advanced suicidal engagement, leveraging a real-world dataset derived from a digital prevention service. We then coupled these predictions with SHAP. By computing additive explanations, we aimed to gain further insight into the language features that most strongly impact the model's classifications. This may be of relevance to clinicians taking decisions informed by the model's output.” |
| **Methods** | | | |
| Data | 5a | D;E | Describe the sources of data separately for the development and evaluation datasets (eg, randomized trial, cohort, routine care or registry data), the rationale for using these data, and representativeness of the data  “We obtained chat transcripts from krisenchat”  “We collected transcripts, a form of routine data, from sessions …”  “To date the service stands as one of the most used and leading mental health prevention services for youth in Germany. ” |
|  | 5b | D;E | Specify the dates of the collected participant data, including start and end of participant accrual; and, if applicable, and of follow-up  “from sessions held between 2021-11-30, and 2022-04-30. ” |
| Participants | 6a | D;E | Specify key elements of the study setting (eg, primary care, secondary care, general population) including the number and location of centers  “mental health prevention services for youth in Germany. ” |
|  | 6b | D;E | Describe the eligibility criteria for study participants  We collected transcripts, a form of routine data, from sessions held between 2021-11-30, and 2022-04-30. We excluded sessions from individuals under 14 years old and those without age information. Those individuals were either unable to give informed consent or wouldn't comply with research standards.  We excluded any sessions that were too short to assess the presence of suicidal ideation and behaviors (SIB) or that ended before counseling began. We handed over the remaining sessions to expert raters. Out of 14,073 sessions, we found 3,193 control sessions, 2,886 of which met our criteria for length and age. |
|  | 6c | D;E | Give details of any treatments received, and how they were handled during model development or evaluation, if relevant  N.A. |
| Data preparation | 7 | D;E | Describe any data pre-processing and quality checking, including whether this was similar across relevant sociodemographic groups  “Before analyzing these messages, we removed all personal identifiers. Subsequently, the text data was preprocessed to enhance readability and reduce text variability. We then improved the readability of the text by eliminating links, HTML tags, social media handles, ASCII characters, Unicode block smileys, and special characters beyond typical German punctuation.”  “We performed one-way Analysis of Variance (ANOVA) tests to evaluate differences in word usage and age across three groups: non-suicidal, suicidal ideation, and advanced suicidal engagement.” |
| Outcome | 8a | D;E | Clearly define the outcome that is being predicted and the time horizon, including how and when assessed, the rationale for choosing this outcome, and whether the method of outcome assessment is consistent across sociodemographic groups  Suicidal ideation that involves actual lethal means indicates a progression to an acquired capability for suicide, leading to a shift towards suicidal actions. Consequently, we have categorized the Columbia-Suicide Severity Rating Scale (C-SSRS) items "Wish to be Dead" and "Nonspecific Active Suicidal Thoughts" under the sub-category of Suicidal Ideation(SI). We have also grouped "Active Suicidal Ideation with Any Methods (Not Plan) without Intent to Act," "Active Suicidal Ideation with Some Intent to Act, without Specific Plan," "Active Suicidal Ideation with Specific Plan and Intent," and actual attempts of suicide—clearly suicidal behavior—into the sub-category of Advanced Suicidal Engagement(ASE).  The Suicidal Ideation category includes those who may simply express a desire not to wake up anymore to those who clearly state they want to end their lives. The Advanced Suicidal Engagement category encompasses individuals contemplating specific lethal methods such as medication or razor blades, those setting a definite date for their action, and those writing farewell letters. It also includes cases where individuals are found just before or right after engaging in potentially fatal behaviors, such as standing on a bridge or openly discussing their immediate plans to consume lethal doses of a substance. |
|  | 8b | D;E | If outcome assessment requires subjective interpretation, describe the qualifications and demographic characteristics of the outcome assessors  N.A. |
|  | 8c | D;E | Report any actions to blind assessment of the outcome to be predicted  N.A. |
| Predictors | 9a | D | Describe the choice of initial predictors (eg, literature, previous models, all available predictors) and any pre-selection of predictors before model building  N.A. |
|  | 9b | D;E | Clearly define all predictors, including how and when they were measured (and any actions to blind assessment of predictors for the outcome and other predictors)  N.A. |
|  | 9c | D;E | If predictor measurement requires subjective interpretation, describe the qualifications and demographic characteristics of the predictor assessors  N.A. |
| Sample size | 10 | D;E | Explain how the study size was arrived at (separately for development and evaluation), and justify that the study size was sufficient to answer the research question. Include details of any sample size calculation  See Sample allocation diagram below  Sample Size calculation and or power estimations may not be applicable to deep neural networks |
| Missing data | 11 | D;E | Describe how missing data were handled. Provide reasons for omitting any data  we excluded sessions from individuals under 14 years old and those without age information. Those individuals were either unable to give informed consent or wouldn't comply with research standards. |
| Analytical methods | 12a | D | Describe how the data were used (eg, for development and evaluation of model performance) in the analysis, including whether the data were partitioned, considering any sample size requirements  To test model stability, we performed repeated cross-validation. We initialized random seeds, then shuffled and split the data (75% for training, 25% for validation). In each round, we maintained consistent class ratios for both training and testing through stratified shuffling. We also applied random oversampling to both sets individually to ensure balanced class ratios. |
|  | 12b | D | Depending on the type of model, describe how predictors were handled in the analyses (functional form, rescaling, transformation, or any standardization)  The input text is encoded into a multidimensional array, then padded or truncated to fit a maximum of 50 tokens, arranged in session arrays of up to 75 messages. After tokenization, the encoder processes the input, producing a 768-dimensional embedding that captures the contextual meaning of the sentences. |
|  | 12c | D | Specify the type of model, rationale†, all model building steps, including any hyperparameter tuning, and method for internal validation  See Statistical analysis section |
|  | 12d | D;E | Describe if and how any heterogeneity in estimates of model parameter values and model performance was handled and quantified across clusters (eg, hospitals, countries). See TRIPOD-Cluster for additional considerations‡  N.A. |
|  | 12e | D;E | Specify all measures and plots used (and their rationale) to evaluate model performance (eg, discrimination, calibration, clinical utility) and, if relevant, to compare multiple models  see Evaluation and Cross-Validation section. |
|  | 12f | E | Describe any model updating (eg, recalibration) arising from the model evaluation, either overall or for particular sociodemographic groups or settings  N.A. |
|  | 12g | E | For model evaluation, describe how the model predictions were calculated (eg, formula, code, object, application programming interface)  The model outputs a probability value between 0 and 1. |
| Class imbalance | 13 | D;E | If class imbalance methods were used, state why and how this was done, and any subsequent methods to recalibrate the model or the model predictions  In each round, we maintained consistent class ratios for both training and testing through stratified shuffling. We also applied random oversampling to both sets individually to ensure balanced class ratios. |
| Fairness | 14 | D;E | Describe any approaches that were used to address model fairness and their rationale  N.A. |
| Model output | 15 | D | Specify the output of the prediction model (eg, probabilities, classification). Provide details and rationale for any classification and how the thresholds were identified  The model will output a probability between 0 and 1 for each output class. |
| Training versus evaluation | 16 | D;E | Identify any differences between the development and evaluation data in healthcare setting, eligibility criteria, outcome, and predictors  It is randomly sampled from the same dataset. |
| Ethical approval | 17 | D;E | Name the institutional research board or ethics committee that approved the study and describe the participant informed consent or the ethics committee waiver of informed consent.  Ethics Approval: Ethikkommission der International Psychoanalytic University (IPU) Berlin, 2023_08 |
| **Open science** | | | |
| Funding | 18a | D;E | Give the source of funding and the role of the funders for the present study  no particular finding for this study |
| Conflicts of interest | 18b | D;E | Declare any conflicts of interest and financial disclosures for all authors  see conflicts of interest section |
| Protocol | 18c | D;E | Indicate where the study protocol can be accessed or state that a protocol was not prepared  was not prepared |
| Registration | 18d | D;E | Provide registration information for the study, including register name and registration number, or state that the study was not registered  no registration was made. |
| Data sharing | 18e | D;E | Provide details of the availability of the study data  due to privacy concerns we cannot make any data public |
| Code sharing | 18f | D;E | Provide details of the availability of the analytical code  see reference to the github repository |
| **Patient and public involvement** | | | |
| Patient and public involvement | 19 | D;E | Provide details of any patient and public involvement during the design, conduct, reporting, interpretation, or dissemination of the study or state no involvement  N.A. |
| **Result** | | | |
| Participants | 20a | D;E | Describe the flow of participants through the study, including the number of participants with and without the outcome and, if applicable, a summary of the follow-up time. A diagram may be helpful  See Participant Flow diagram. |
|  | 20b | D;E | Report the characteristics overall and, where applicable, for each data source or setting, including the key dates, key predictors (including demographics), treatments received, sample size, number of outcome events, follow-up time, and amount of missing data. A table may be helpful. Report any differences across key demographic groups  See characteristics in table 3. |
|  | 20c | E | For model evaluation, show a comparison with the development data of the distribution of important predictors (demographics, predictors, and outcome) |
| Model development | 21 | D;E | Specify the number of participants and outcome events in each analysis (eg, for model development, hyperparameter tuning, model evaluation)  We started with 2,311 rated sessions and selected 1,348 complete and eligible sessions for detailed analysis. We used 1,011 cases for training and 337 for testing. You can find detailed class compositions and sample demographics in (Multimedia Appendix Table 3). After balancing the classes and resampling, the training set had 1,254 cases, and the test set had 420 cases. |
| Model specification | 22 | D | Provide details of the full prediction model (eg, formula, code, object, application programming interface) to allow predictions in new individuals and to enable third party evaluation and implementation, including any restrictions to access or reuse (eg, freely available, proprietary)¶  The script can be accessed on GitHub at the following location: [https://github.com/krisenchat/p91-suicide-model/]. |
| Model performance | 23a | D;E | Report model performance estimates with confidence intervals, including for any key subgroups (eg, sociodemographic). Consider plots to aid presentation  The transformer model, which performed the best, achieved an overall accuracy of 0.79, 95% CI[0.73, 0.99], and a macro-averaged Area Under the Curve (AUC) of 0.89, 95% CI[0.81, 0.91]. In contrast, the baseline word2vec model achieved an overall accuracy of 0.61, 95% CI[0.61, 0.80], and a macro-averaged AUC of 0.77, 95% CI[0.64, 0.90]. |
|  | 23b | D;E | If examined, report results of any heterogeneity in model performance across clusters. See TRIPOD-Cluster for additional details‡  N.A. |
| Model updating | 24 | E | Report the results from any model updating, including the updated model and subsequent performance  N.A. |
| **Discussion** | | | |
| Interpretation | 25 | D;E | Give an overall interpretation of the main results, including issues of fairness in the context of the objectives and previous studies  See p.20  Calibration metrics revealed significant reliability issues for both models, suggesting a need for recalibration before clinical use. Due to these calibration concerns, the clinical utility of the models should be interpreted cautiously, as small sample sizes introduce more noise to neural networks [3].  Performance improvements over the baseline may be overstated due to less emphasis on feature engineering in the word-vector model. Incorporating n-grams, tf-idf, or dictionary-based features could address the lack of sequential text structure in word embeddings, which this study did not fully tackle.  There might also be age or gender biases in the expression of suicidality, as the sample was not gender-balanced, potentially skewing results towards female expressions of suicidal ideation and behaviors. Class imbalance required the use of oversampling techniques, which might introduce biases. Technical limitations led to session truncations, affecting data quality and representativeness. |
| Limitations | 26 | D;E | Discuss any limitations of the study (such as a non-representative sample, sample size, overfitting, missing data) and their effects on any biases, statistical uncertainty, and generalisability  There might also be age or gender biases in the expression of suicidality, as the sample was not gender-balanced, potentially skewing results towards female expressions of suicidal ideation and behaviors. Class imbalance required the use of oversampling techniques, which might introduce biases. Technical limitations led to session truncations, affecting data quality and representativeness.  Calibration metrics revealed significant reliability issues for both models, suggesting a need for recalibration before clinical use. Due to these calibration concerns, the clinical utility of the models should be interpreted cautiously, as small sample sizes introduce more noise to neural networks [3]. |
| Usability of the model in the context of current care | 27a | D | Describe how poor quality or unavailable input data (eg, predictor values) should be assessed and handled when implementing the prediction model  N.A. |
|  | 27b | D | Specify whether users will be required to interact in the handling of the input data or use of the model, and what level of expertise is required of users  N.A. |
|  | 27c | D;E | Discuss any next steps for future research, with a specific view to applicability and generalisability of the model  Despite the promise of this study, future research could enhance the model's accuracy and utility. Developing dynamic models that track changes over time and include additional factors like demographics could improve performance. This could be achieved by I.e. using retrained transformers in extracting longitudinal event data [4]or by incorporating explanations into the clinicians counseling environment [5]. Integrating transformer embeddings into multimodal graph network models might refine precision and bolster prevention efforts by handling the multifactorial nature of suicide. |

### Consort-AI checklist

The following article checklist is part of the consort checklist, which was first published by Collins and Colleagues[6]. Items are marked in black, and responses and citations are in blue.

**Table S3.** Consort-AI checklist.

| Item | Description | Addressed in Manuscript |
| --- | --- | --- |
| 1a | Identification as a study of AI intervention(s) | Partially addressed. The title mentions "Explainable Artificial Intelligence Text Classifier" but doesn't explicitly state it's a study of AI intervention. |
| 1b | Structured summary of trial design, methods, results, and conclusions | Addressed. The abstract provides a structured summary including background, objective, methods, results, and conclusions. |
| 2a | Scientific background and explanation of rationale | Addressed. The introduction provides background on suicide prevention and the potential of AI in this field. |
| 2b | Specific objectives or hypotheses | Addressed. The study aims and hypotheses are clearly stated at the end of the introduction. |
| 3a | Description of trial design | Partially addressed. The study design is described, but it's not a trial per se. |
| 3b | Important changes to methods after trial commencement | Not applicable. This is not a trial. |
| 4a | Eligibility criteria for participants | Addressed. Inclusion criteria are described in the Methods section. |
| 4b | Settings and locations where the data were collected | Addressed. The setting (krisenchat, a German crisis intervention service) is described. |
| 5 | The interventions for each group with sufficient details to allow replication | Partially addressed. The AI models are described, but this is not an intervention study. |
| 6a | Completely defined pre-specified primary and secondary outcome measures | Addressed. The outcome measures (suicidal ideation and advanced suicidal engagement) are defined. |
| 6b | Any changes to trial outcomes after the trial commenced | Not applicable. This is not a trial. |
| 7a | How sample size was determined | Not explicitly addressed. The sample size is given, but the rationale for this size is not explained. |
| 7b | When applicable, explanation of any interim analyses and stopping guidelines | Not applicable. |
| 8a | Method used to generate the random allocation sequence | Not applicable. This is not a randomized study. |
| 8b | Type of randomisation; details of any restriction | Not applicable. |
| 9 | Mechanism used to implement the random allocation sequence | Not applicable. |
| 10 | Who generated the random allocation sequence, who enrolled participants, and who assigned participants to interventions | Not applicable. |
| 11a | If done, who was blinded after assignment to interventions and how | Not applicable. |
| 11b | If relevant, description of the similarity of interventions | Not applicable. |
| 12a | Statistical methods used to compare groups for primary and secondary outcomes | Addressed. Statistical analysis methods are described. |
| 12b | Methods for additional analyses, such as subgroup analyses and adjusted analyses | Addressed. Additional analyses like SHAP are described. |
| 13a | For each group, the numbers of participants who were randomly assigned, received intended treatment, and were analysed for the primary outcome | Partially addressed. The number of sessions analyzed is provided, but this is not a randomized trial. |
| 13b | For each group, losses and exclusions after randomisation, together with reasons | Not applicable. |
| 14a | Dates defining the periods of recruitment and follow-up | Partially addressed. The date range for data collection is provided. |
| 14b | Why the trial ended or was stopped | Not applicable. |
| 15 | A table showing baseline demographic and clinical characteristics for each group | Addressed. Demographic information is provided in Appendix Table 2. |
| 16 | For each group, number of participants (denominator) included in each analysis and whether the analysis was by original assigned groups | Partially addressed. The number of sessions in each group is provided. |
| 17a | For each primary and secondary outcome, results for each group, and the estimated effect size and its precision | Addressed. Results for each model and outcome are provided with confidence intervals. |
| 17b | For binary outcomes, presentation of both absolute and relative effect sizes is recommended | Not applicable. The outcomes are not binary. |
| 18 | Results of any other analyses performed, including subgroup analyses and adjusted analyses, distinguishing pre-specified from exploratory | Addressed. Additional analyses like SHAP are reported. |
| 19 | All important harms or unintended effects in each group | Not applicable. This is not an intervention study. |
| 20 | Trial limitations, addressing sources of potential bias, imprecision, and, if relevant, multiplicity of analyses | Addressed. Limitations are discussed in detail. |
| 21 | Generalisability (external validity | applicability) of the trial findings |
| 22 | Interpretation consistent with results, balancing benefits and harms, and considering other relevant evidence | Addressed. The discussion provides interpretation consistent with the results. |
| 23 | Registration number and name of trial registry | Not applicable. This is not a registered trial. |
| 24 | Where the full trial protocol can be accessed, if available | Not applicable. |
| 25 | Sources of funding and other support, role of funders | Partially addressed. Conflicts of interest are stated, but funding sources are not explicitly mentioned. |

**References**

1. Collins GS, Reitsma JB, Altman DG, Moons KGM. Transparent Reporting of a multivariable prediction model for Individual Prognosis or Diagnosis (TRIPOD): the TRIPOD statement. *Ann Intern Med*. 2015;162(1):55-63. doi:10.7326/M14-0697
2. Boudreaux ED, Rundensteiner E, Liu F, et al. Applying Machine Learning Approaches to Suicide Prediction Using Healthcare Data: Overview and Future Directions. *Front Psychiatry*. 2021;12. doi:10.3389/fpsyt.2021.707916
3. Reeves KW, Vasconez G, Weiss SJ. Characteristics of Suicidal Ideation: A Systematic Review. *Arch Suicide Res Off J Int Acad Suicide Res*. 2022;26(4):1736-1756. doi:10.1080/13811118.2021.2022551
4. Gipson PY, Agarwala P, Opperman KJ, Horwitz A, King CA. Columbia-Suicide Severity Rating Scale. *Pediatr Emerg Care*. 2015;31(2):88-94. doi:10.1097/PEC.0000000000000225
5. Joiner T. *Why People Die by Suicide*. Harvard University Press; 2005.
6. Collins GS, Moons KGM, Dhiman P, et al. TRIPOD+AI statement: updated guidance for reporting clinical prediction models that use regression or machine learning methods. *BMJ*. 2024;385:e078378. doi:10.1136/bmj-2023-078378
