# Supplementary material for: An Explainable Artificial Intelligence Text Classifier for Suicidality Prediction in Youth Crisis Text Line Users: Development and Validation Study
Source: JMIR Public Health Surveill. 2025 Jan 29;11:e63809. doi: 10.2196/63809 (PMC11822322; doi:10.2196/63809)
Supplement: Multimedia Appendix 2 [file publichealth_v11i1e63809_app2.docx]

**Table S1.** Parameters and their values used in the Transformer–MLP**^a^** and W2V-MLP**^b^**.

| **Model Name** | **T-MLP Parameter** | **T-MLP^a^** | **W2V-MLP**  **Parameter** | **W2V-MLP^b^** |
| --- | --- | --- | --- | --- |
| Encoder | Model name | *XLM-RoBERTa-base* | fasttext cc.de.300.bin | fasttext cc.de.300.bin |
|  | Max. tokens per message | 50 | Input text length | 400 |
|  | Max. messages per input | 75 |  |  |
|  | Embedding dimension  (per message) | 768 | embedding dimension  (per word) | 300 |
|  | Vocabulary size |  | vocabulary size | 30,000 |
| Decoder | Time Distributed Units | 100 | CNN^c^ Filters | 15 |
|  | BiLSTM^d^ Units | 2*25 | BiLSTM^d^ Units | 2*15 |
| Hyperparameters | Dropout | 0.5 | Dropout | 0.5 |
|  | Batch size | 10 | Batch size | 10 |
|  | Activation | ReLu^e^ | Activation | ReLu^e^ |
|  | Optimizer | AdamW^f^ | Optimizer | AdamW^f^ |

^a^Transformer-Multi-Layer-Perceptron

^b^Word2Vector-Multilayer-Perceptron

^c^Convolutional Neural Network

^d^Bidirectional Long-Short-Term-Memory-Gate

^e^Rectified Linear Unit

^f^Adaptive Moment Estimation optimizer with Weight Decay

**Table S2.** linguistic and demographic characteristics of the sample.

| measure | N | Mean (SD) | 95% CI |
| --- | --- | --- | --- |
| number of words |  |  |  |
| total | 1,348 | 327.71 (265.60) | [313.52, 341.90] |
| NS^a^ | 500 (37.1%) | 409.71 (308.65) | [382.58, 436.83] |
| SI^b^ | 558 (41.4%) | 252.39 (205.69) | [235.29, 269.50] |
| ASE^c^ | 290 (21.5%) | 252.39 (205.69) | [302.91, 359.61] |
| age in years |  |  |  |
| total | 1,348 | 17.94 (3.17) | [17.77, 18.11] |
| NS^a^ | 500 (37.1%) | 18.23 (3.23) | [17.95, 18.52] |
| SI^b^ | 558 (41.4%) | 17.92 (3.14) | [17.66, 18.18] |
| ASE^c^ | 290 (21.5%) | 17.48 (3.07) | [17.12, 17.83] |

^a^ non-suicidal

^b^ Suicidal Ideation

^c^ Advanced Suicidal Engagement

^d^SD: standard deviation

**Table S3.** Brier score decomposition and model comparison.

|  | **transformer** | | | **word2vec** | | |
| --- | --- | --- | --- | --- | --- | --- |
| **class** | NS^a^ | SI^b^ | SB^c^ | NS | SI | SB |
| **reliability** | 0.002 | 0.007 | 0.008 | 0.001 | 0.010 | 0.011 |
| **resolution** | 0.219 | 0.863 | 0.106 | 0.06 | 0.015 | 0.023 |
| **uncertainty** | 0.222 | 0.222 | 0.222 | 0.222 | 0.222 | 0.222 |
| **average Brier loss** | 0.10 | | | 0.18 | | |
| **Brier Skill Score** | 44.4% | | | | | |

^a^ non-suicidal

^b^ Suicidal Ideation

^c^ Advanced Suicidal Engagement

## **Figure**


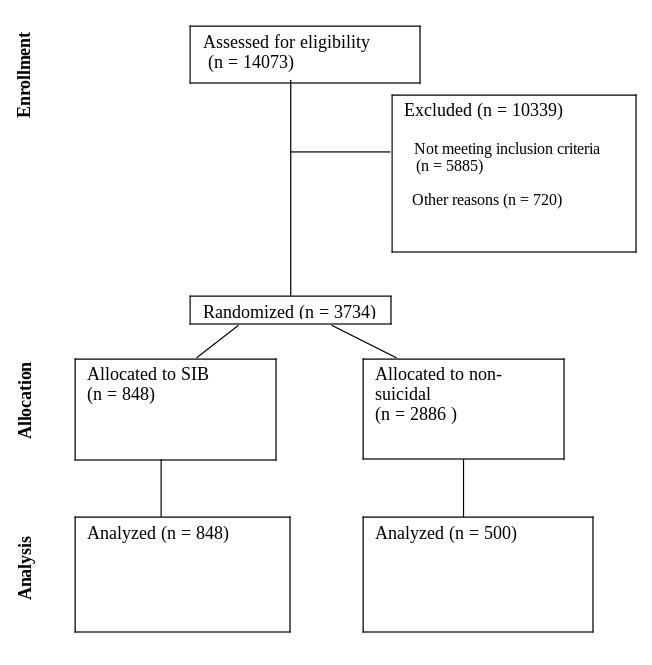


**Figure S1.** Consort participant flow, sampling, and inclusion diagram.
