# Supplementary material for: An Explainable Artificial Intelligence Text Classifier for Suicidality Prediction in Youth Crisis Text Line Users: Development and Validation Study
Source: JMIR Public Health Surveill. 2025 Jan 29;11:e63809. doi: 10.2196/63809 (PMC11822322; doi:10.2196/63809)
Supplement: Multimedia Appendix 3 [file publichealth_v11i1e63809_app3.docx]

## **A Primer on Neural Networks and Metrics**

Unlike traditional statistical inference methods, neural networks iteratively estimate the parameters—or weights—of a function that maps input data to an output, according to the outcome, which serves as the supervising element within this data-driven input-output mapping. While neural networks may share underlying algorithms with regression, how parameters are estimated differs significantly. Conceptually, a neural network can be considered a series of parallel regressions passed through mathematical filters to identify complex patterns in the data.

During the training phase, the input data is repeatedly presented to the network in cycles known as epochs. Each epoch involves one complete presentation of the data samples to the learning algorithm. The primary objective during training is to identify the weights that minimize prediction errors quantified by a loss function tailored to the specific prediction task. This loss typically represents the average prediction error across all predictions in a validation dataset.

Error reduction is achieved through gradient descent, where the algorithm incrementally adjusts weights to decrease loss. In each step, the algorithm evaluates whether slightly increasing or decreasing a weight will reduce the loss and adjusts the weight accordingly.

Ultimately, the neural network is expected to converge to a solution that minimizes loss as effectively as possible. However, when predicting a classification task, the model will output a floating value between 0 and 1, commonly called confidence, for the desired output. A prediction threshold is then defined to determine whether a sample is classified as a positive or negative instance of an event. Typically, this threshold is set at 0.5 but is subject to threshold tuning, as predictions will inform decisions about the event in question. This process is crucial because the model may either overpredict or underpredict the event based on its learned weights. Sometimes, this behavior might be desirable depending on the inherent bias of what the model has learned from the training set. For instance, we should avoid unnecessary intervention costs if the model is biased toward flagging samples as positive instances. However, this procedure assumes that the probabilities are meaningful and can be interpreted as genuine confidence values only if the model did not overfit or underfit the training data. This means the model could extract meaningful parameters without overly emphasizing specifics of the training set that do not generalize to the actual distribution of data it was supposed to estimate.

## **Training Curves**

Training curves are invaluable tools used to illustrate the learning process of an algorithm, typically in the context of neural networks, throughout all training epochs. Training generally occurs on a designated subset of the data known as the training set, which is used to estimate the model's parameters. A holdout dataset—often called a validation set—is employed to gauge the model's ability to generalize to unseen data. This step is crucial because it prevents the model from memorizing the training data. It may result in perfect predictions on those samples but potentially poor prediction performance on new, real-world data.

To visualize the model's learning progress, average loss and accuracy metrics are calculated after each epoch for both the training and validation sets. These metrics are then plotted on a two-dimensional graph with epochs on the x-axis and the values of loss and accuracy on the y-axis. Ideally, the plot will show the loss decreasing asymptotically towards zero and the accuracy increasing asymptotically towards one, indicating a 100% correct classification rate. However, due to factors such as statistical noise, poor data quality, or a limited number of training samples, achieving such perfect metrics is often not feasible.

Training curves also help identify trends such as overfitting, which is indicated when the training loss decreases significantly while the validation loss begins to increase. This suggests that the model is overly specialized to the training data, compromising its ability to perform well on new, unseen data.


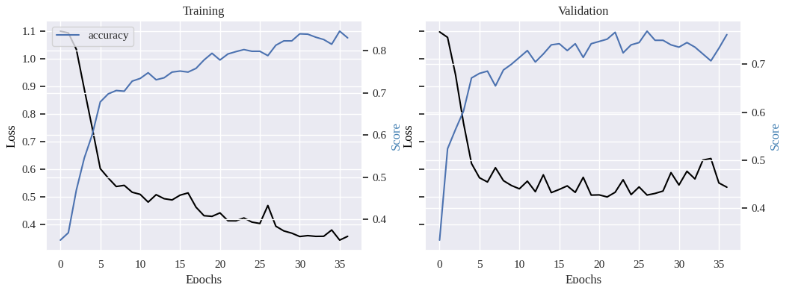


**Figure S1.** Learning curves of the transformer-MLP, loss in black, accuracy in blue over the course of training epochs on the x-axis. Training is set on the left, and validation is set on the right. The learning curve shows smooth and steady learning.


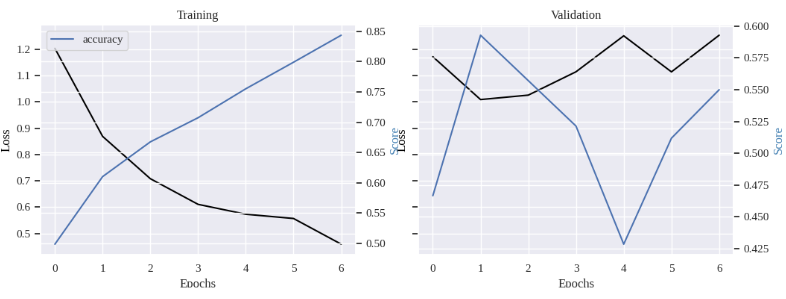


**Figure S2.** Learning Curves of the Transformer-MLP, loss in black, accuracy in blue over the course of training epochs on the x-axis. The depiction of the training set is on the left, and the validation set is on the right. The curve shows considerable training instability.

## **Decision Curve Analysis**

A model generated by a neural network can be conceptualized as a clinical test, where decision thresholds dictate the classification of outcomes. These decisions are inherently imperfect due to the model's reliance on limited information, leading to four possible outcomes: true positive, false positive, true negative, and false negative. Sensitivity and specificity measure the model's ability to identify positive and negative instances correctly.

Decision-making in clinical settings involves weighing the costs associated with incorrect decisions, such as the severe consequences of missing a critical diagnosis or the burdens of unnecessary interventions. The trade-off between sensitivity and specificity implies that no test is without cost. Evaluating a model based solely on accuracy, sensitivity, and specificity may not assess its clinical utility adequately. Decision curves, therefore, provide a valuable method for evaluating the trade-offs across various decision thresholds, illustrating the net benefit and helping to determine the optimal balance between true positives and false positives.

Threshold tuning is essential in this process. By adjusting the level at which the model classifies an instance as positive or negative, clinicians can fine-tune the model's sensitivity to triggering alarms, directly affecting its clinical utility.

However, the net benefit should only be inspected across some probabilities. Setting a reasonable threshold range is crucial. This threshold is determined by the risk we are willing to accept in a clinical, real-world decision scenario. The range should be clinically informed, considering the consequences of false decisions. For instance, in cases of potential suicide attempts, where the consequences of missing an attempt are severe, a lower threshold may be preferred to ensure a more sensitive classifier.

### **Practical Computation**

For the computation, we define the following constructs:

*Net Benefit(NB):* Calculated as the true positives minus the false positives, weighted by the threshold probability (see formula below). The goal is to determine whether a predictive model provides more correct than incorrect prognoses, factoring in the clinical significance of correct predictions over false ones.

*Threshold Probability(p):* This is the probability at which the decision to treat or not treat is equivocal, reflecting the severity of missing a true positive individual relative to the consequences of unnecessary treatment of a false positive individual.

1. set a probability: For each threshold probability in small increments, we obtain predictions
2. assess the number of TP and FP
3. The net benefit is then computed as follows:

$$NB = \frac{TP}{N} - \frac{FP(\frac{p}{1-p})}{N}$$

N is the total number of individuals in the validation sample, and $\frac{p}{1-p}$ the harm-to-benefit ratio is obtained by taking the odds of the threshold probability.

1. Compute the Net Benefit of Treat-All and Treat-None
2. Defined as the true positive rate minus the false positive rate multiplied by the odds of the threshold probability.$TA = 1 - \frac{FP(\frac{p}{1-p})}{N}$
3. Here, no instances are classified as positive, resulting in a net benefit of zero.

TN = 0

The obtained values are plotted in a two-dimensional graph, with NB on the y-axis and threshold probabilities on the x-axis.


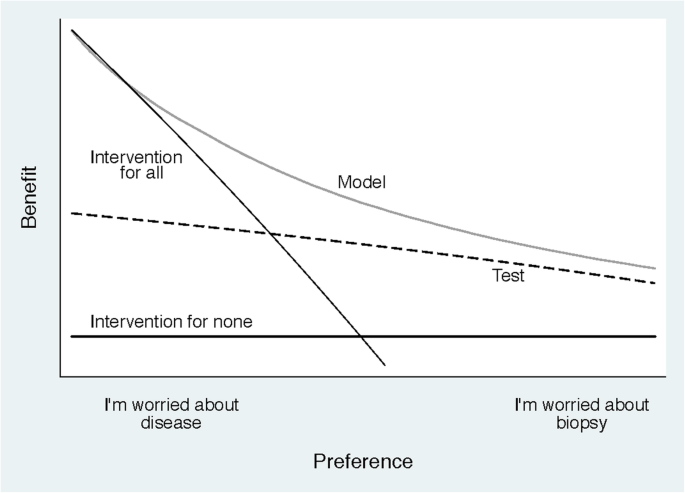


**Figure S3.** A prototypical Decision Curve, extracted from Vickers and colleagues [1]: A simple, step-by-step guide to interpreting decision curve analysis p.3

### **Interpretation**

The model’s clinical utility is assessed by comparing its net benefit to that of "Treat All" or "Treat None" decision strategies across the clinically relevant threshold probabilities. A higher net benefit in lower threshold ranges suggests a sensitive model prone to more false positives, which may be acceptable if the cost of missing a case is high. Conversely, a model that performs well at higher threshold probabilities tends to be more specific, reducing the cost of unnecessary interventions.

A model is considered clinically useful as a decision support tool if its net benefit is higher than the "Treat All" or "Treat None" strategies. Ideally, it should outperform any other comparative model across the chosen threshold probabilities. The net benefit (NB) can theoretically range between negative infinity and 1. For example, a net benefit of 0.2 represents the detection of 20 true positives (TP) for every 100 individuals in the target population without an increase in false positives (FP). This absence of an increase in FP is attributed to how the NB is computed. The selected probability threshold corresponds to the so-called harm-to-benefit (htb) ratio, which expresses how many FPs we are willing to treat unnecessarily for each TP. For instance, in scenarios with severe outcomes, such as suicide attempts, treating 99 FPs for each TP may be justified. The risk and costs of the treatment are other important factors when it comes to selecting a ratio. For example, a ratio of 1:99 corresponds via the odds function to a threshold probability of 1 percent, calculated as $Odds = \frac{0.01}{1-0.01}=\frac{0.01}{0.99}=\frac{1}{99}$

### **Reading Further**

A range of excellent articles describes the rationale and interpretation of decision curves:
1) Decision curve analysis: a novel method for evaluating prediction models [2]

2) Reporting and Interpreting Decision Curve Analysis: A Guide for Investigators [3]

3) A simple, step-by-step guide to interpreting decision curve analysis [1]

4) A website that even holds an excellent software tutorial on the computation of DCA in several statistical programming languages! [4]

### **Reliability Analysis Using Calibration Curves**

Reliability is the degree to which the result of a measurement, test, calculation, or specification can be depended on to be accurate. It expresses whether and how well a test measures what it is intended to measure.

In the context of Machine Learning, reliability may be operationally defined as the calibration of a model. Calibration assesses how well a classifier's probabilistic predictions correspond to the actual outcomes. This is particularly important for probabilistic models in healthcare or finance, where decisions are based on the predicted probabilities from classifiers. For example, a medical diagnosis system may determine a patient's disease risk based on symptoms and test results.

Proper calibration is essential for accurately interpreting key test metrics like sensitivity and specificity, which are influenced by the classifier's biases. A miscalibrated model will over- or underestimate the outcome, potentially rendering it unreliable or unsafe in clinical settings. Consequently, calibration influences all other metrics with which the predicted outcome is assessed, including clinical utility. Moreover, miscalibration could lead to overestimating or underestimating the net benefit of a decision at a certain threshold. Calibration is, therefore, crucial for trusting clinical utility results.

Only with near-perfect calibration can the probabilities output by a neural network be considered true reflections of prediction certainty. If a model is poorly calibrated, techniques such as Platt scaling or isotonic regression can be employed to recalibrate the predicted probabilities and improve reliability.

## **Calibration Curves**

Calibration curves, also known as reliability diagrams, plot predicted probabilities against the actual probabilities (1 or 0). For a well-calibrated model, these predictions should ideally align with the diagonal line of the graph, indicating that the predicted probability matches the observed frequency of outcomes. This alignment demonstrates whether a classifier's predictions, made with high confidence, genuinely correspond to a higher likelihood of accuracy.

Calibration curves can also expose biases in the model's predictions across different subgroups or output probability ranges. For instance, a model might consistently overestimate the risk of an event for one group and underestimate it for another. Identifying these biases is crucial for correcting them, enhancing the fairness and effectiveness of machine learning models across diverse scenarios.

### **Practical Computation**

1. Collect Data: Gather predicted probabilities and actual outcomes (0 or 1) from the validation set.
2. Bin Grouping: Sort the probabilities and group them into specified intervals (e.g., evenly spaced segments of 0.1).
3. Calculate Statistics per Bin:
4. *Average Predicted Probability:* Compute the mean of the predicted probabilities within each bin.
5. *Observed Frequency:* Calculate the ratio of positive instances (true outcomes) to total instances in the bin.
6. Plot the Calibration Curve: The x-axis represents the average predicted probability, and the y-axis shows the observed frequency.


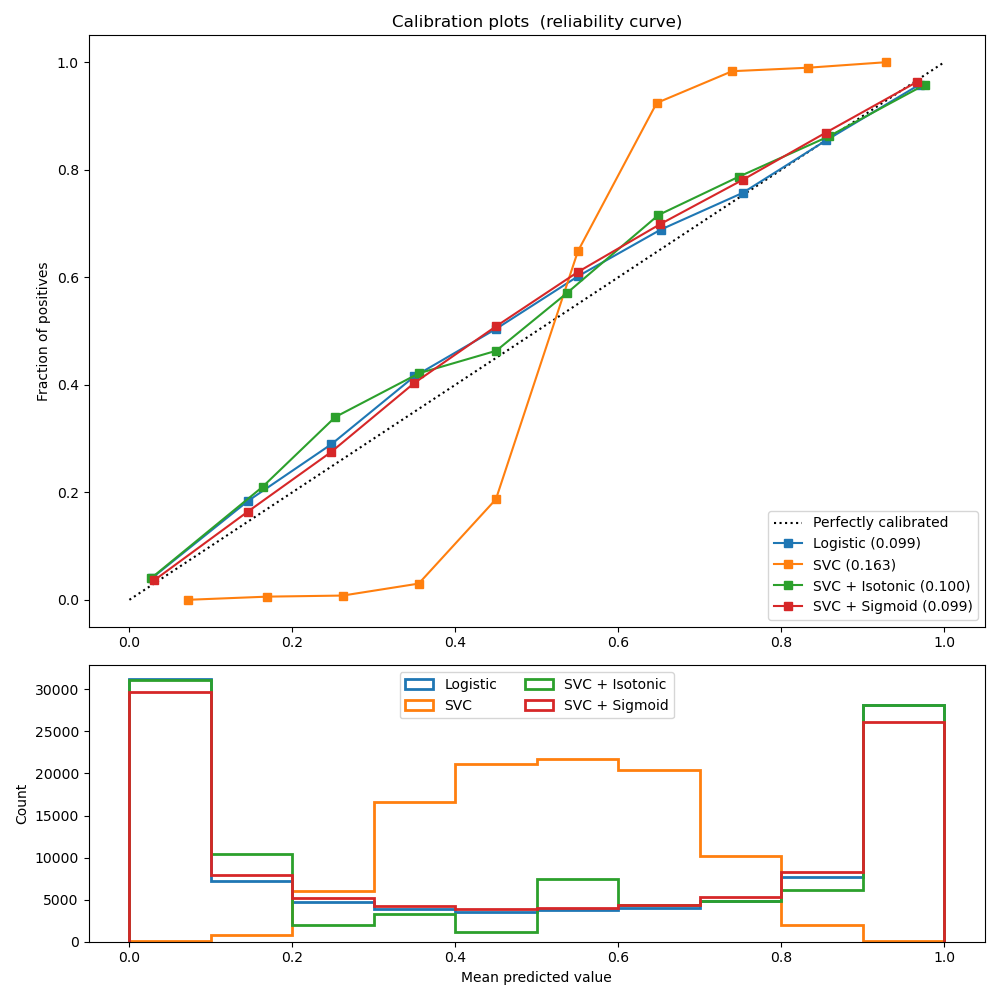


**Figure S4.** A prototypical reliability diagram from the sci-kit learn documentation [5]**.**

### **Interpretation**

A perfectly calibrated model produces a calibration curve that closely aligns with the diagonal line, where the predicted probability equals the observed frequency. The closer the curve to this diagonal, the better the calibration. Deviations indicate miscalibration:

1. *Above the Diagonal:* The model is under-confident; actual outcomes are more frequent than predicted.
2. *Below the Diagonal:* The model is over-confident; actual outcomes are less frequent than predicted.

## **Explainable AI with Shapley Additive Explanations**

### **Introduction to Explainable AI (XAI)**

Explainable AI (XAI) refers to techniques that enhance the transparency and interpretability of machine learning models for human understanding. The primary objective of XAI is to bridge the gap between the high performance of complex AI models, such as deep neural networks, and the need for humans to comprehend the decision-making or prediction processes. XAI methods encompass a range of approaches, including feature importance metrics, model visualizations, simpler surrogate models, Local Interpretable Model-agnostic Explanations (LIME), and Shapley Additive Explanations (SHAP).

The importance of XAI in fields such as healthcare, finance, and legal systems cannot be overstated. Understanding the rationale behind AI-driven decisions is crucial for ensuring ethical use, maintaining regulatory compliance, and fostering trust among stakeholders. XAI techniques enable practitioners to validate model behavior, detect biases, and ensure alignment with domain expertise and ethical guidelines.

### **Post-Hoc Explainability**

Post-hoc explainability models are applied after a model has been trained and is actively making predictions. These methods aim to elucidate the model's decisions without modifying internal mechanisms, providing insights into the decision-making process. Specifically, post-hoc methods reveal which modeled features have been more influential in the prediction process.

Post-hoc explainability is particularly valuable when working with complex, "black-box" models such as deep neural networks. It allows researchers and practitioners to gain insights into model behavior without sacrificing the predictive power of sophisticated algorithms. This approach is crucial in scenarios where model performance is paramount, but interpretability is also required for regulatory, ethical, or practical reasons.

### **Computation of Shapley Values**

Shapley values, originating from cooperative game theory, have been adapted to quantify the contribution of each feature in a machine learning model's prediction. This method, known as SHAP (SHapley Additive exPlanations), assigns an importance value to each feature, indicating its contribution relative to a baseline prediction.

The computation of Shapley values involves considering all possible combinations of features and assessing how each feature's presence or absence affects the model's output. This process ensures a fair attribution of importance to each feature, considering complex interactions between features.

#### ***Calculation Process:***

1. *Define the Baseline:* Typically, the average prediction across the dataset or a subset of background data.
2. *Iterate Over Feature Combinations:* This might include combinations of word tokens, excluding the feature (token) under consideration for models involving text.
3. *Compute Contributions:* Calculate the difference in prediction with and without the feature and sum these differences across all possible combinations. This sum is the Shapley value, representing the feature's average marginal contribution.
4. *Repeat for All Features:* This process is replicated for each feature to understand its impact.

#### ***Interpretation***

Shapley values can be interpreted similarly to other feature importance metrics:

Negative Values: Indicate a feature’s influence towards a negative prediction (0).

Positive Values: Suggest a feature’s influence towards a positive prediction (1).

Visualization: These contributions can be visualized using bar plots, beeswarm plots, or inline text highlighting, where colors (e.g., red for positive, blue for negative) can denote the contribution of word tokens.

Some words of caution need to be spoken about using Shapley values in combination with transformer models: Transformers, characterized by their deep architecture and contextual embeddings, present unique challenges for Shapley values:

- Contextual Dependencies: Unlike simpler models, transformers' features (tokens) are not independent but contextually dependent on surrounding words. This makes isolating the contribution of single tokens complex.
- Dynamic Semantics: The meaning and, therefore, the contribution of a word can change with its context, a nuance that Shapley values might only capture with sophisticated modeling.
- Potential Risks of Misinterpretation
- Complex Interpretations: The deep integration of information in transformers can obscure individual feature contributions, potentially reducing details about decisions.
- Overinterpretation: SHAP's simplified explanations may lead users to overlook the model's complexities and limitations, potentially resulting in misguided decisions, particularly in critical areas like healthcare or legal applications.

#### ***Conclusion***

Applying SHAP to transformer models with contextual embeddings requires careful consideration of the method's limitations and the model's inherent complexities. Stakeholders should be cautious and critically evaluate SHAP-based explanations, especially when making significant decisions based on these insights.

#### ***Reading Further***

1. Open access chapter on the fundamentals of explainable AI: [6]
2. The original Shapley additive explanations method paper: [7]
3. Gitbook on explainable machine learning, chapter dedicated to SHAP: [8]

References

1. Vickers AJ, Calster B, Steyerberg EW. A simple, step-by-step guide to interpreting decision curve analysis. *Diagn Progn Res*. 2019;3(1). doi:10.1186/s41512-019-0064-7
2. Vickers AJ, Elkin EB. Decision curve analysis: a novel method for evaluating prediction models. *Med Decis Mak Int J Soc Med Decis Mak*. 2006;26(6):565-574. doi:10.1177/0272989X06295361
3. Van Calster B, Wynants L, Verbeek JFM, et al. Reporting and Interpreting Decision Curve Analysis: A Guide for Investigators. *Eur Urol*. 2018;74(6):796-804. doi:10.1016/j.eururo.2018.08.038
4. DCA: Homepage. Accessed July 30, 2024. https://mskcc-epi-bio.github.io/decisioncurveanalysis/index.html
5. Probability Calibration curves — scikit-learn 0.24.2 documentation. Accessed July 30, 2024. https://scikit-learn.org/0.24/auto_examples/calibration/plot_calibration_curve.html
6. Holzinger A, Saranti A, Molnar C, Biecek P, Samek W. Explainable AI Methods - A Brief Overview. In: Holzinger A, Goebel R, Fong R, Moon T, Müller KR, Samek W, eds. *xxAI - Beyond Explainable AI: International Workshop, Held in Conjunction with ICML 2020, July 18, 2020, Vienna, Austria, Revised and Extended Papers*. Springer International Publishing; 2022:13-38. doi:10.1007/978-3-031-04083-2_2
7. Lundberg S, Lee SI. A Unified Approach to Interpreting Model Predictions. *Publ Online Novemb*. 2017;24. doi:10.48550/arXiv.1705.07874
8. Molnar C. *9.6 SHAP (SHapley Additive exPlanations) | Interpretable Machine Learning*. Accessed July 30, 2024. https://christophm.github.io/interpretable-ml-book/shap.html
